# Thermally Conductive Ti_3_C_2_T_*x*_ Fibers with Superior Electrical Conductivity

**DOI:** 10.1007/s40820-025-01752-x

**Published:** 2025-04-27

**Authors:** Yuxiao Zhou, Yali Zhang, Yuheng Pang, Hua Guo, Yongqiang Guo, Mukun Li, Xuetao Shi, Junwei Gu

**Affiliations:** Shaanxi Key Laboratory of Macromolecular Science and Technology, School of Chemistry and Chemical Engineering, Northwestern Polytechnical University, Xi’an, 710072 People’s Republic of China

**Keywords:** Thermally conductive Ti_3_C_2_T_*x*_ fibers, Interlayer crosslinking, High electrical conductivity, Density functional theory simulation, Equilibrium molecular dynamics simulation

## Abstract

High-performance Ti_3_C_2_T_*x*_ fibers have garnered significant potential for smart fibers enabled fabrics. Nonetheless, a major challenge hindering their widespread use is the lack of strong interlayer interactions between Ti_3_C_2_T_*x*_ nanosheets within fibers, which restricts their properties. Herein, a versatile strategy is proposed to construct wet-spun Ti_3_C_2_T_*x*_ fibers, in which trace amounts of borate form strong interlayer crosslinking between Ti_3_C_2_T_*x*_ nanosheets to significantly enhance interactions as supported by density functional theory calculations, thereby reducing interlayer spacing, diminishing microscopic voids and promoting orientation of the nanosheets. The resultant Ti_3_C_2_T_*x*_ fibers exhibit exceptional electrical conductivity of 7781 S cm^−1^ and mechanical properties, including tensile strength of 188.72 MPa and Young’s modulus of 52.42 GPa. Notably, employing equilibrium molecular dynamics simulations, finite element analysis, and cross-wire geometry method, it is revealed that such crosslinking also effectively lowers interfacial thermal resistance and ultimately elevates thermal conductivity of Ti_3_C_2_T_*x*_ fibers to 13 W m^−1^ K^−1^, marking the first systematic study on thermal conductivity of Ti_3_C_2_T_*x*_ fibers. The simple and efficient interlayer crosslinking enhancement strategy not only enables the construction of thermal conductivity Ti_3_C_2_T_*x*_ fibers with high electrical conductivity for smart textiles, but also offers a scalable approach for assembling other nanomaterials into multifunctional fibers.

**Supplementary Information:**

The online version contains supplementary material available at 10.1007/s40820-025-01752-x.

## Highlights

- The strong covalent crosslinking between trace amounts of borates and the hydroxyl groups of Ti_3_C_2_T_*x*_ significantly reduces interlayer spacing, enhances orientation and compactness, leading to notable improvements in both the mechanical (tensile strength of 188.72 MPa) and electrical properties (7781 S cm^−1^) of Ti_3_C_2_T_*x*_ fibers.
- Trace amounts of borates can promote the regularization of interfacial structures, reduce interfacial thermal resistance, and significantly enhance the thermal conductivity (13 W m^−1^ K^−1^) of Ti_3_C_2_T_*x*_ fibers, thus increasing their potential for efficient heat transfer applications.

**Supplementary Information:**

The online version contains supplementary material available at 10.1007/s40820-025-01752-x.

## Graphical abstract

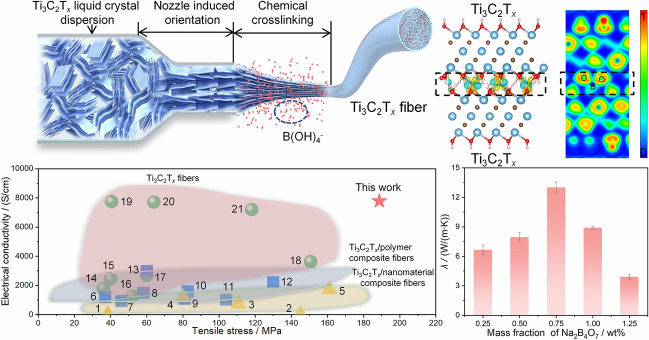

## Introduction

The rapid development of wearable devices, flexible electronics, and aerospace has highlighted the growing challenges of signal delay and overheating in high-performance electronics, making it difficult for existing fibers to meet the dual needs of fast signal transmission and efficient heat dissipation [1, 2]. Consequently, novel fibers are urgently needed to seamlessly integrate high electrical and thermal conductivity to enhance the signal transmission efficiency and stability of advanced electronics [3–5]. Ti_3_C_2_T_*x*_, as a novel two-dimensional nanomaterial, has emerged as an ideal candidate for producing high-performance fibers due to its exceptional electrical conductivity, thermal conductivity, and mechanical properties [6–8].

Researchers have primarily focused on incorporating Ti_3_C_2_T_*x*_ with polymers or other nanomaterials to fabricate Ti_3_C_2_T_*x*_ composite fibers using scalable wet spinning techniques [9–11]. Gu et al*.* [12] employed wet spinning to prepare Ti_3_C_2_T_*x*_/polyrotaxane composite fibers, which demonstrated the best overall performance when the mass fraction of Ti_3_C_2_T_*x*_ was 85 wt%, including a tensile strength of 188.7 MPa and an electrical conductivity of 247.5 S cm^−1^. He et al*.* [13] fabricated Ti_3_C_2_T_*x*_/reduced graphene oxide composite fibers via wet spinning, having optimal tensile strength (110.7 MPa) and electrical conductivity (743.1 S cm^−1^) with the Ti_3_C_2_T_*x*_ content of 60 wt%. Although the addition of polymers and other nanomaterials enhances the mechanical properties of Ti_3_C_2_T_*x*_ composite fibers, their inherent non-conductivity or poor conductivity, along with the interface mismatch with Ti_3_C_2_T_*x*_, induces significant electron scattering that severely hampers electron transport [14–16]. As a result, the failure of the composite fibers to achieve the anticipated breakthrough in electrical conductivity occurs despite a high loading of Ti_3_C_2_T_*x*_ [17, 18].

Studies have shown that the issues of introducing nonconductive materials and interface mismatch can be effectively avoided through the fabrication of Ti_3_C_2_T_*x*_ fibers by sole assembling Ti_3_C_2_T_*x*_ nanosheets, which enable a breakthrough in the electrical conductivity of Ti_3_C_2_T_*x*_ fibers [19, 20]. However, the weak interlayer interactions between Ti_3_C_2_T_*x*_ nanosheets pose a significant challenge of poor mechanical properties and easy brittleness for Ti_3_C_2_T_*x*_ fibers during the assembly process [21, 22]. Some efforts have been made to enhance the mechanical properties of Ti_3_C_2_T_*x*_ fibers by reinforcing interlayer connectivity with the hydrogen and ionic bonding [23, 24]. Zhang et al*.* [24] used an acetic acid aqueous solution as a coagulation bath, in which hydrogen bonding formed by acetic acid and Ti_3_C_2_T_*x*_ nanosheets improved interlayer interactions between the nanosheets, resulting in fibers with good electrical conductivity (4048 S cm^−1^) and certain mechanical properties (tensile strength < 10 MPa). However, the insufficient interlayer interactions from hydrogen and ionic bonding give rise to multiple defects within Ti_3_C_2_T_*x*_ fibers, including excessive interlayer spacing, microscale porosity, and local disorder of the nanosheets, which constrain the mechanical properties and hinder further enhancement of their electrical conductivity [25, 26]. Furthermore, although Ti_3_C_2_T_*x*_ is theoretically known for its outstanding thermal conductivity, little research can be conducted on the thermal properties of Ti_3_C_2_T_*x*_ fibers [6, 27]. Given the stringent thermal conductivity requirements of high-performance electronics [28, 29], it is crucial for in-depth exploration of the thermal conductivity in Ti_3_C_2_T_*x*_ fibers to address this research gap and facilitate their widespread use in practical electrical and thermal conductivity applications [30, 31].

Compared to hydrogen and ionic bonding, covalent bonds formed through electron sharing offer stronger binding forces and greater structural stability, which are expected to reduce the interlayer spacing, minimize microscopic porosity, and enhance the orientation of the nanosheets, thereby simultaneously improving the mechanical properties, electrical and thermal conductivity for the fibers [32–34]. In nature, the plant cell wall is reinforced by the covalent crosslinking of trace amounts of borate with the hydroxymethyl groups of pectin, which helps enhance the mechanical properties of the wall and strengthen the intercellular network [35, 36]. Building on this inspiration, we propose a strategy of interfacial covalent crosslinking to design and fabricate high-performance Ti_3_C_2_T_*x*_ fibers, where regulating the concentration of Ti_3_C_2_T_*x*_ liquid-crystalline dispersion and the strong covalent crosslinking between borate and the hydroxyl groups on the Ti_3_C_2_T_*x*_ surface enables efficient and continuous assembly of Ti_3_C_2_T_*x*_ fibers. Based on the density functional theory (DFT) calculations, the mechanism by which trace amounts of borate enhance the interlayer interactions, thus improving the interlayer spacing, and promoting the orientation and densification within Ti_3_C_2_T_*x*_ fibers is systematically investigated. The intrinsic factors contributing to the significant enhancement of both electrical conductivity and mechanical properties for Ti_3_C_2_T_*x*_ fibers through the optimization of the nanosheet microstructure are thoroughly discussed. A combination of simulation and experimental validation, including equilibrium molecular dynamics (EMD) simulations, finite element analysis, and crossline testing methods, is employed to deeply analyze the impact of borate content on the thermal conductivity of Ti_3_C_2_T_*x*_ fibers.

## Experimental Section

### Materials

Ti_3_AlC_2_ powder (particle size ≈400 mesh) was obtained from 11 Technology Co., Ltd. (Jilin, China). Sodium tetraborate decahydrate (Na_2_B_4_O_7_·10H_2_O), concentrated hydrochloric acid (HCl), lithium fluoride (LiF), and ethyl alcohol were all purchased from Shanghai Macklin Biochemical Technology Co., Ltd. (Shanghai, China).

### Preparation of Ti_3_C_2_T_x_ Fibers via Wet Spinning

Firstly, 25 mg mL^−1^ of Ti_3_C_2_T_*x*_ liquid-crystalline dispersion was prepared and placed in the syringe as spinning solution. The Ti_3_C_2_T_*x*_ spinning solution was passed through the nozzle with a diameter of 200 μm under a syringe pump pushing speed of 3.6 mL h^−1^ and extruded into the rotary coagulation bath (rotating speed of 9.42 mm s^−1^). The coagulants consisted of aqueous/ethanol (7:3 vol/vol) solutions containing different mass fractions of Na_2_B_4_O_7_ (0.25, 0.50, 0.75, 1.00, and 1.25 wt%). The Ti_3_C_2_T_*x*_ gel fibers were formed immediately upon contact of the spinning solution with the coagulation bath and were continuously produced as the coagulation bath was rotated. The Ti_3_C_2_T_*x*_ gel fibers were soaked in the coagulation bath for 10 min and then transferred to a washing solution consisting of deionized water and ethanol (7:3 vol/vol). The washed Ti_3_C_2_T_*x*_ gel fibers were collected on a drum and dried in air to obtain Ti_3_C_2_T_*x*_ fibers (B content in Ti_3_C_2_T_*x*_ fibers is given in Table S1), which were stored in a drying chamber.

## Results and Discussion

### Characterization of Ti_3_C_2_T_***x***_ Dispersion

**Fig. 1:**
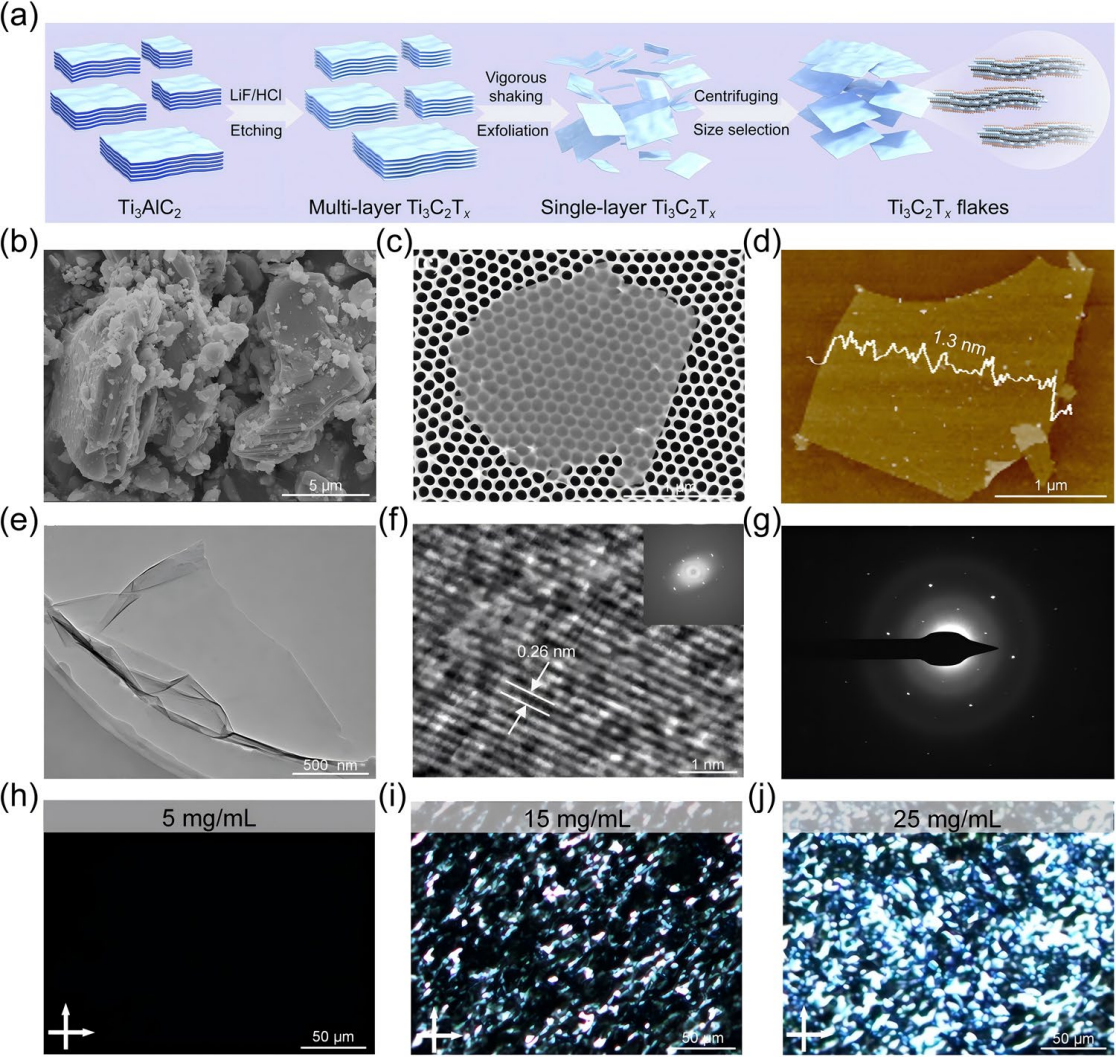
Preparation and characterization of Ti_3_C_2_T_*x*_ nanosheets and dispersion. **a** Schematic for fabrication of Ti_3_C_2_T_*x*_. **b** SEM image of Ti_3_AlC_2_. **c** SEM, **d** AFM, **e** TEM, **f** HR-TEM and its FFT diffraction pattern (the inset), and **g** SAED images of Ti_3_C_2_T_*x*_. POM images of Ti_3_C_2_T_*x*_ dispersion at concentrations of **h** 5 mg mL^−1^, **i** 15 mg mL^−1^, and **j** 25 mg mL^−1^

The schematic illustration of the preparation for Ti_3_C_2_T_*x*_ nanosheets is shown in Fig. 1a. The scanning electron microscope (SEM) image of Ti_3_AlC_2_ (Fig. 1b) displays its typical layered structure. After selective etching with LiF and HCl, followed by mechanical oscillation and differential centrifugation, the obtained Ti_3_C_2_T_*x*_ nanosheets show an average lateral size of approximately 2.47 ± 0.69 μm, according to SEM image (Fig. 1c) and size distribution (Fig. S2) of Ti_3_C_2_T_*x*_ nanosheets [37]. Moreover, Fig. 1d displays the atomic force microscopy (AFM) image of Ti_3_C_2_T_*x*_ nanosheets with a thickness approximately 1.3 nm and a length-to-width ratio (l/d) greater than 10^3^, confirming the successful preparation of single-layer Ti_3_C_2_T_*x*_ nanosheets [38]. The transmission electron microscopy (TEM) image of Ti_3_C_2_T_*x*_ (Fig. 1e) shows that the nanosheets are highly transparent and free of impurities, which indicates the absence of by-products. The single-layer Ti_3_C_2_T_*x*_ nanosheets also maintain a well-ordered crystal structure, as evidenced by clear lattice fringes with a *d*-spacing of 0.26 nm corresponding to the (100) plane observed in the high-resolution TEM (HR-TEM) image (Fig. 1f) [39]. Additionally, the fast Fourier transform (FFT) pattern in the inset of Fig. 1f shows typical hexagonal diffraction spots, which align with the selected area electron diffraction (SAED) pattern (Fig. 1g), further indicating the single-crystal structure of Ti_3_C_2_T_*x*_with hexagonal atomic arrangement [40]. Furthermore, the successful removal of the Al layer in Ti_3_C_2_T_*x*_ after strong acid etching is confirmed by the disappearance of the (104) peak belonging to Ti_3_AlC_2_ in the X-ray diffraction (XRD) spectrum (Fig. S3a) [41]. As observed from the X-ray photoelectron spectroscopy (XPS) spectrum (Fig. S3b), the Al 2*p* and Al 2*s* peaks associated with Ti_3_AlC_2_ in Ti_3_C_2_T_*x*_ have distinctly disappeared, and a characteristic peak of the F 1*s* appears at 685 eV, indicating that Ti_3_C_2_T_*x*_ primarily contains four elements: Ti, C, O, and F [42].

According to Onsager’s theory, the formation of lyotropic liquid-crystalline phase from two-dimensional nanosheets is governed by the competition between translational and rotational entropy in the dispersion system [43, 44]. The polarized optical microscopy (POM) images of Ti_3_C_2_T_*x*_ dispersion at different concentrations are shown in Fig. 1h-j. At a concentration of 5 mg mL^−1^, Ti_3_C_2_T_*x*_ dispersion shows no birefringence, and thus remains isotropic (Fig. 1h). This is because, at low concentrations, the large free volume results from the relatively low excluded volume, which refers to the space inaccessible due to the presence of Ti_3_C_2_T_*x*_ nanosheets. In this case, it is difficult for Ti_3_C_2_T_*x*_ nanosheets to rotate and align with translational entropy dominating, instead undergoing random motion and disordered distribution. When the concentration of Ti_3_C_2_T_*x*_ dispersion is 15 mg mL^−1^, Ti_3_C_2_T_*x*_ dispersion shows appearing birefringent texture under crossed polarizers (Fig. 1i), performing the formation of a nematic liquid-crystalline phase. This is attributed to the increase in excluded volume and corresponding decrease in free volume, which favors rotational entropy. As a result, Ti_3_C_2_T_*x*_ nanosheets begin to align locally and promote the formation of the liquid-crystalline phase. Furthermore, the birefringent texture becomes highly obvious and uniformly distributed at a concentration of 25 mg mL^−1^ (Fig. 1j), signaling that Ti_3_C_2_T_*x*_ dispersion has completely transitioned from isotropic to anisotropic. At this point, the internal nanosheets are locally aligned without aggregation, which provides a unique advantage for the assembly of Ti_3_C_2_T_*x*_ fibers with aligned nanosheets in the wet spinning process. The rheological properties of Ti_3_C_2_T_*x*_ liquid-crystalline dispersion at a concentration of 25 mg mL^−1^ are investigated in Fig. S4. The viscosity of Ti_3_C_2_T_*x*_ liquid-crystalline dispersion decreases with increasing shear rate, displaying shear thinning non-Newtonian behavior, which thus allows the dispersion to flow continuously throughout the nozzle during extrusion (Fig. S4a). Moreover, the significantly higher storage modulus (G′) compared to the loss modulus (G′′) across the entire frequency range (Fig. S4b) indicates the gel-like behavior of Ti_3_C_2_T_*x*_ liquid-crystalline dispersion, enabling it to retain its shape after extrusion and stabilize fiber formation even when shear force is removed [45].

### Fabrication of Ti_3_C_2_T_***x***_ Fibers

**Fig. 2:**
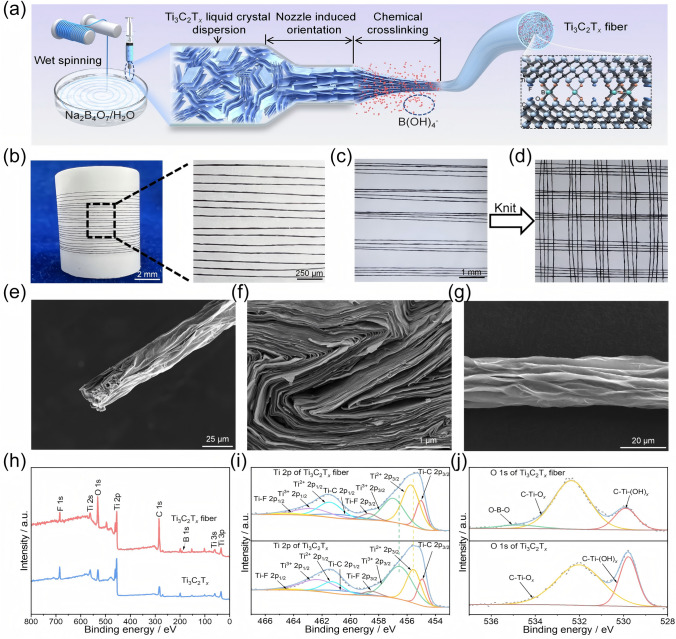
Fabrication, morphology, and chemical characterization of Ti_3_C_2_T_*x*_ fibers. **a** Schematic for fabricating Ti_3_C_2_T_*x*_ fibers. Photographs of Ti_3_C_2_T_*x*_ fibers **b** wound on the bobbin and **c-d** woven. SEM images of Ti_3_C_2_T_*x*_ fibers: **e** overall, **f** cross section, and **g** side-section views. **h** XPS full spectra, and XPS narrow spectra of **i** Ti 2*p* and **j** O 1*s* of Ti_3_C_2_T_*x*_ powder and Ti_3_C_2_T_*x*_ fiber

Figure 2a illustrates the preparation process of Ti_3_C_2_T_*x*_ fibers. Thanks to the excellent dispersibility and rheological properties of Ti_3_C_2_T_*x*_ liquid-crystalline dispersion [46], the spinning solution was successfully extruded through a fine nozzle into a rotating coagulation bath containing Na_2_B_4_O_7_, where interfacial crosslinking facilitated the formation of continuous gel fibers with a stable spinning process, followed by drying to obtain Ti_3_C_2_T_*x*_ fibers, whereas extrusion into a coagulation bath without Na_2_B_4_O_7_ did not result in gel fiber formation (Fig. S5 and Movie S1). The fabricated Ti_3_C_2_T_*x*_ fibers are continuously produced with uniform size (Figs. 2b and S6) and exhibit excellent mechanical properties, allowing for manual weaving into textiles (Fig. 2c, d). The overall SEM image of Ti_3_C_2_T_*x*_ fiber (Fig. 2e) shows an average diameter of 23 μm. Cross-sectional and side-view SEM images of the fiber (Fig. 2f, g) demonstrate that Ti_3_C_2_T_*x*_ nanosheets are highly aligned and densely packed along the fiber axis, suggesting the alignment of locally ordered Ti_3_C_2_T_*x*_ nanosheets due to shear forces from the fine nozzle and interfacial crosslinking during the wet spinning process.

From the XPS spectra of Ti_3_C_2_T_*x*_ and Ti_3_C_2_T_*x*_ fiber in Fig. 2h, Ti_3_C_2_T_*x*_ fiber appears a new characteristic peak at 191.8 eV associated with B 1*s* after wet spinning in addition to the F, Ti, O, and C elements compared to Ti_3_C_2_T_*x*_, suggesting that borate may undergo chemical crosslinking with the hydroxyl groups on the Ti_3_C_2_T_*x*_ surface in Ti_3_C_2_T_*x*_ fiber. Figure 2i reveals that the XPS narrow spectra of Ti 2*p* is mainly composed of two parts, Ti 2*p*_3/2_ and Ti 2*p*_1/2_, each of which can be fitted into four components corresponding to Ti–C, Ti^2+^, Ti^3+^, and Ti-F. The peaks of Ti^2+^ 2*p*_3/2_ and Ti^3+^ 2*p*_3/2_ shift from 455.6 and 456.6 eV for Ti_3_C_2_T_*x*_ to 455.8 and 457.0 eV for Ti_3_C_2_T_*x*_ fiber, respectively. This is because the electron cloud density around Ti atoms is decreased when B atoms in Ti_3_C_2_T_*x*_ fiber form covalent bonds with Ti atoms via borate ester bonds due to the higher electronegativity of B atoms than Ti atoms, thereby confirming the formation of borate ester covalent bonds between the borate and hydroxyl groups on Ti_3_C_2_T_*x*_ nanosheets [47]. This chemical crosslinking disrupts and replaces the electrostatic repulsion between Ti_3_C_2_T_*x*_ nanosheets, which promotes the transition of Ti_3_C_2_T_*x*_ from liquid-crystalline dispersion to macroscopic fibers. Additionally, the formation of borate ester bonds is directly evidenced by the new characteristic peak of O–B–O at 534.8 eV from Ti_3_C_2_T_*x*_ fiber (Fig. 2j). As indicated by the Fourier transform infrared spectroscopy (FTIR) spectra in Fig. S7, the characteristic peaks of Ti_3_C_2_T_*x*_ at 3426 and 1663 cm^−1^ are attributed to the typical stretching vibrations of –OH and C=O groups, whereas the decreased intensity of the –OH peak at 3426 cm^−1^ and the appearance of the B–O characteristic peak at 1168 cm^−1^ in Ti_3_C_2_T_*x*_ fiber further prove the covalent crosslinking between nanosheets via borate ester bonds. The uniform distribution of Ti element and the sparse distribution of B element in the cross section of Ti_3_C_2_T_*x*_ fiber (Fig. S8) further confirm that borate effectively react with the hydroxyl groups on Ti_3_C_2_T_*x*_ nanosheets during the wet spinning process, and the formation for trace amounts of borate ester covalent bonds facilitates the assembly of Ti_3_C_2_T_*x*_ nanosheets into fibers [48].

### Structural Characterization, Mechanical, and Electrical Properties of Ti_3_C_2_T_***x***_ Fibers

**Fig. 3:**
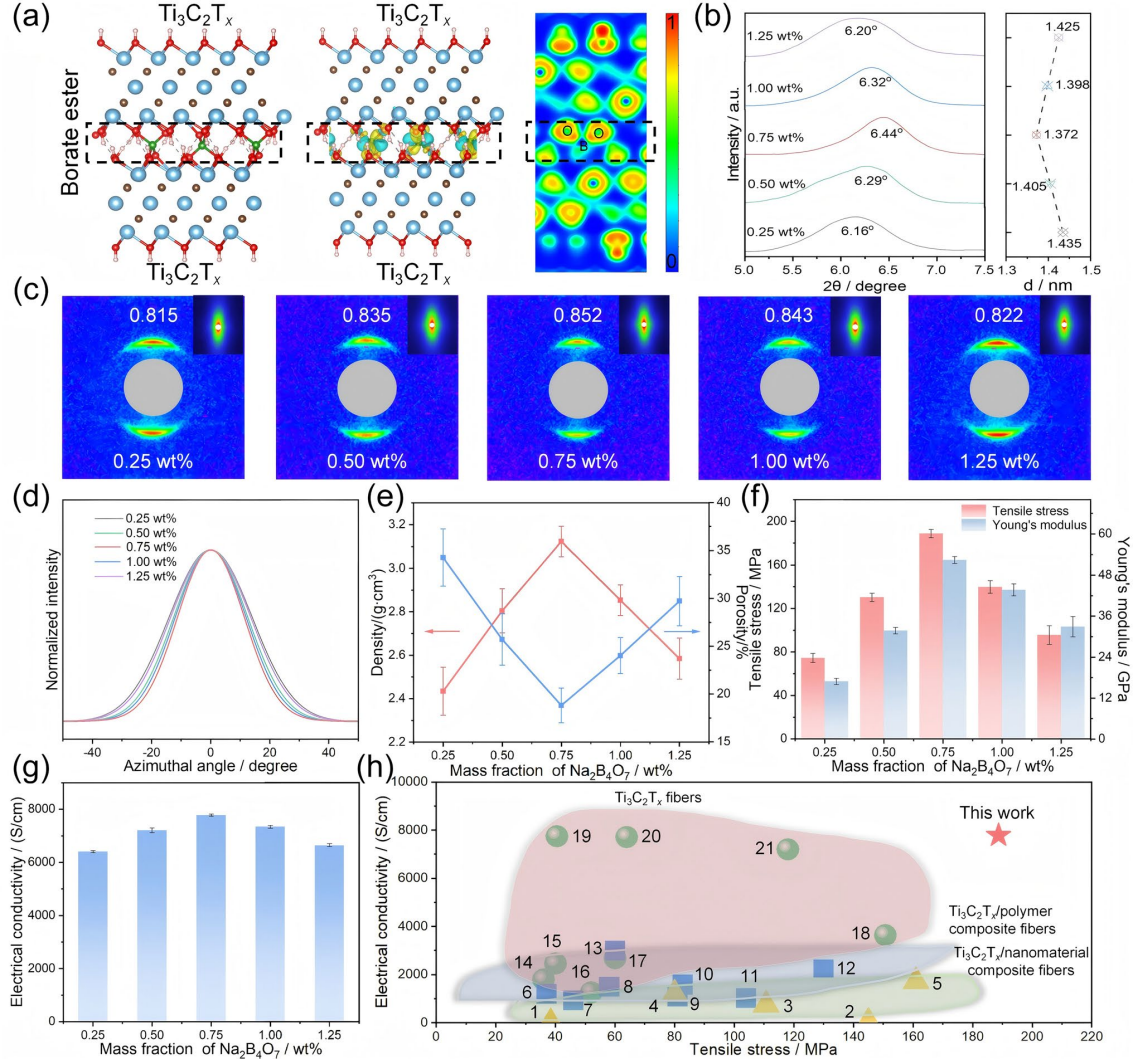
Structural characterization, mechanical, and electrical properties of Ti_3_C_2_T_*x*_ fibers. **a** DFT calculations of borate ester bond between adjacent Ti_3_C_2_T_*x*_ nanosheets. **b** XRD patterns, **c** WAXS and SAXS (inset) patterns graphs, **d** plots of azimuthal angle according to the WAXS patterns, **e** density and porosity, **f** tensile strength and Young’s modulus, and **g** electrical conductivity of Ti_3_C_2_T_*x*_ fibers with different Na_2_B_4_O_7_ contents. **h** Comparisons of electrical conductivity versus tensile strength of Ti_3_C_2_T_*x*_ fibers prepared with 0.75 wt% Na_2_B_4_O_7_ against reported Ti_3_C_2_T_*x*_-based fibers and Ti_3_C_2_T_*x*_ fibers

DFT calculations (Fig. 3a) further elucidate the interfacial crosslinking induced by covalent interactions in Ti_3_C_2_T_*x*_ fibers. The optimized structure of the borate ester bond between adjacent Ti_3_C_2_T_*x*_ nanosheets (left) is derived from DFT [49, 50]. The charge density difference (CDD) illustrates the changes in charge density at the interface (middle), where the decreased and increased charge densities are represented in cyan and yellow, respectively [51]. It can be observed that the increase in charge density is primarily localized in the interatomic region between the O atoms on the Ti_3_C_2_T_*x*_ nanosheets and the B atoms introduced by Na_2_B_4_O_7_, confirming the formation of B–O covalent bonds. Besides, the B–O bond length between adjacent Ti_3_C_2_T_*x*_ nanosheets is relatively short, ranging from 1.47 to 1.61 Å, which aids the formation of a stable B–O tetrahedral structure between the nanosheets. The electron localization function (ELF) diagram (right) shows the degree of electron localization at the interface, with fully red (1) regions indicating complete localization and fully blue (0) regions indicating no localization [52]. Notably, significant electron localization is shown between the O and B atoms, further corroborating the strong nature of the B–O bond. The XRD patterns of Ti_3_C_2_T_*x*_ fibers (Fig. 3b) show the (002) characteristic peaks at 6.16°, 6.29°, 6.44°, 6.32°, and 6.20° for *c* of 0.25, 0.50, 0.75, 1.00, and 1.25 wt%, respectively. The corresponding average interlayer spacing (d-spacing) was calculated using Bragg’s law [53], revealing a trend where the d-spacing of the fibers first decreases and then increases as the Na_2_B_4_O_7_ content rises. The covalent crosslinking gradually eliminates the electrostatic repulsion between adjacent Ti_3_C_2_T_*x*_ nanosheets with the increase of Na_2_B_4_O_7_ content from 0.25 to 0.75 wt%, which leads to a decline of d-spacing from 14.35 to 13.72 Å. However, when the Na_2_B_4_O_7_ content increases from 0.75 to 1.25 wt%, the d-spacing expands from 13.72 to 14.25 Å. This expansion is attributed to the excess borate ester bonds that mitigate the electrostatic repulsion, but also act as intercalation impurities, causing the increase in the spacing between Ti_3_C_2_T_*x*_ nanosheets. Moreover, according to the wide-angle X-ray scattering (WAXS), small-angle X-ray scattering (SAXS) patterns (Fig. 3c), and the corresponding plots of azimuthal angle based on WAXS patterns (Fig. 3d) [54], the full width at half maximum (FWHM) of the (002) peak decreases and the orientation orders enhance from 0.815 to 0.852 with the increment of Na_2_B_4_O_7_ from 0.25 to 0.75 wt% (Fig. S9). This demonstrates that the stable B–O tetrahedral structure formed between Ti_3_C_2_T_*x*_ nanosheets effectively improves the alignment and promotes high orientation of the nanosheets. However, as the Na_2_B_4_O_7_ content further increases to 1.25 wt%, the FWHM of Ti_3_C_2_T_*x*_ fibers broadens with the orientation order reducing to 0.822, suggesting that excess borate ester covalent bonds hinder the effective alignment of the nanosheets within the fibers. The diameters of Ti_3_C_2_T_*x*_ fibers are decreased and then increased with increasing Na_2_B_4_O_7_ (Fig. S10), with the smallest diameter at 0.75 wt% Na_2_B_4_O_7_ due to the reduced d-spacing and enhanced orientation order of Ti_3_C_2_T_*x*_ nanosheets. As shown in Fig. 3e, the porosity of Ti_3_C_2_T_*x*_ fibers is significantly reduced to 18.81%, so as to achieve a high density of 3.12 g cm^−3^ with the increment of Na_2_B_4_O_7_ to 0.75 wt%, indicating a reduction in interlayer porosity and a denser arrangement of the fibers (Fig. S11). Na_2_B_4_O_7_ with more than 0.75 wt% may lead to increased wrinkles and interlayer voids of Ti_3_C_2_T_*x*_ nanosheets, resulting in higher fiber porosity, as evidenced by the increasing intensity of the SAXS pattern (Fig. S12) [40]. Therefore, Ti_3_C_2_T_*x*_ fibers exhibit optimal orientation and density with 0.75 wt% Na_2_B_4_O_7_, which significantly contributes to enhancing the overall performance of the fibers.

As shown in Figs. 3f and S13, the tensile strength and Young’s modulus of Ti_3_C_2_T_*x*_ fibers exhibit an initial increase followed by a decrease as the Na_2_B_4_O_7_ content increases. When the Na_2_B_4_O_7_ content is 0.25 wt%, the tensile strength and Young’s modulus of Ti_3_C_2_T_*x*_ fibers are 74.40 MPa and 16.91 GPa. It is evident that the dissociation of borate ester covalent bonds dissipates substantial energy during stretching, while the formation of these bonds entitles mechanical properties of Ti_3_C_2_T_*x*_ fibers. As Na_2_B_4_O_7_ content increases to 0.75 wt%, Ti_3_C_2_T_*x*_ fibers offer the highest tensile strength and Young’s modulus, 1.54 and 2.10 times higher than those of fibers with 0.25 wt% Na_2_B_4_O_7_, respectively. This improvement is linked to the augmented borate ester bonds acting as bridging sites, which not only enhance the interactions between adjacent nanosheets, but also simultaneously improve the orientation and density of the nanosheets, thereby promoting optimal load transfer across the nanosheets [55]. However, an excess of borate ester bonds may lead to the aggregation of Ti_3_C_2_T_*x*_ nanosheets when the Na_2_B_4_O_7_ content exceeds 0.75 wt%. This aggregation causes stress to concentrate at weak points, including irregular accumulations and voids, which impedes the transfer of stress between nanosheets, thus significantly diminishing tensile strength and Young’s modulus of Ti_3_C_2_T_*x*_ fibers. Moreover, the electrical conductivity of Ti_3_C_2_T_*x*_ fibers initially increases and then decreases with increasing Na_2_B_4_O_7_ content, reaching a peak of 7781 S cm^−1^ with 0.75 wt% Na_2_B_4_O_7_ (Fig. 3g). This is because that the reduced d-spacing and compact stacking of Ti_3_C_2_T_*x*_ nanosheets improve interlayer contact efficiency and support the formation of continuous electron transport pathways. Furthermore, the highly oriented arrangement enables more efficient electron movement between the nanosheets, reducing electron scattering, thereby boosting the electrical conductivity of the fibers. Above all, in comparison to those of previously reported Ti_3_C_2_T_*x*_-based composite fibers and other Ti_3_C_2_T_*x*_ fibers, the resulting macroscopic Ti_3_C_2_T_*x*_ fibers prepared exhibited the highest properties in both tensile strength and conductivity, as summarized in Fig. 3h and Table S2.

### Thermal Conductivity of Ti_3_C_2_T_***x***_ Fibers

**Fig. 4:**
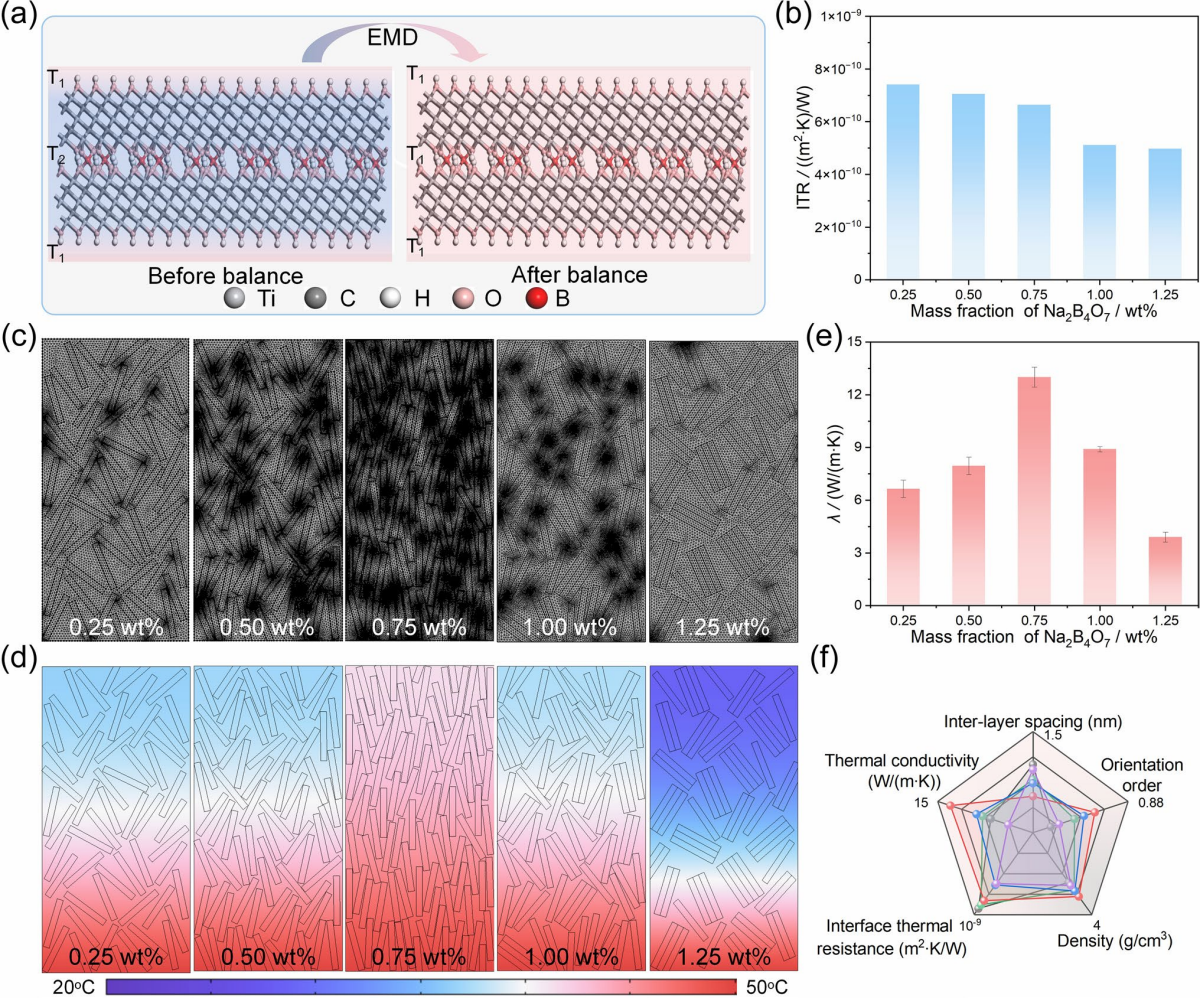
ITR between Ti_3_C_2_T_*x*_ nanosheets and thermal conductivity of Ti_3_C_2_T_*x*_ fibers. **a** Schematic illustration of EMD simulation of borate ester covalently bonded Ti_3_C_2_T_*x*_ nanosheets. **b** ITR between borate ester covalently bonded Ti_3_C_2_T_*x*_ nanosheets with different Na_2_B_4_O_7_ contents obtained by EMD simulation. **c** Extremely fine grid divisions corresponding to the finite element analysis models of Ti_3_C_2_T_*x*_ fibers with different Na_2_B_4_O_7_ contents. **d** Temperature distribution of Ti_3_C_2_T_*x*_ fibers with different Na_2_B_4_O_7_ contents under the same heating temperature and time simulated by finite element analysis. **e** *λ* of Ti_3_C_2_T_*x*_ fibers with different Na_2_B_4_O_7_ contents. **f** Star-plot of the d-spacing, orientation order, density, ITR, and *λ* of Ti_3_C_2_T_*x*_ fibers with different Na_2_B_4_O_7_ contents

The effect of borate ester covalent bonds on the thermal conductivity of Ti_3_C_2_T_*x*_ fibers was explored using both simulation and experimental methods. EMD simulation based on the Green–Kubo method quantified the interfacial thermal resistance (ITR) of Ti_3_C_2_T_*x*_ nanosheets linked by these bonds by building different models with varying Na_2_B_4_O_7_ contents (Fig. S14) [56]. Figure 4a illustrates the EMD schematic for the borate ester bonded Ti_3_C_2_T_*x*_ nanosheets, where the system reaches equilibrium when the heat current auto correlation function approaches zero and the temperature remains stable at 300 K. The total energy in these models of different Na_2_B_4_O_7_ contents was analyzed to assess the corresponding ITR (Fig. S15). The relationship between the ITR of borate ester bonded Ti_3_C_2_T_*x*_ nanosheets and the concentration of Na_2_B_4_O_7_ shows a decrease in ITR as Na_2_B_4_O_7_ content increases (Fig. 4b). Specifically, the ITR of Ti_3_C_2_T_*x*_ nanosheets decreases from 7.413 × 10^−10^ to 4.973 × 10^−10^ m^2^ K W^−1^ when the Na_2_B_4_O_7_ content is raised from 0.25 to 1.25 wt%. The reason is that the high crystalline structure and metallic electrical properties of Ti_3_C_2_T_*x*_ nanosheets allow phonons and electrons to serve effectively as thermal carriers for heat transfer. Most importantly, increasing the Na_2_B_4_O_7_ content results in a more regular interfacial structure between borate ester bonded Ti_3_C_2_T_*x*_ nanosheets, which enhances the directional movement of phonons and electrons, reducing scattering and lowering ITR. Conversely, the interfacial structure becomes disordered, a phenomenon that leads to severe phonon and electron scattering, increasing the ITR when Na_2_B_4_O_7_ content is reduced [57]. At a Na_2_B_4_O_7_ concentration of 0.75 wt%, the ITR is significantly reduced, reaching as low as 6.640 × 10^−10^ m^2^ K W^−1^. Furthermore, finite element analysis was utilized to simulate the temperature distribution of Ti_3_C_2_T_*x*_ fibers with different Na_2_B_4_O_7_ mass fractions under the same heating conditions (bottom temperature of 50 °C for 10 ns) [58], investigating the impact of ITR, d-spacing, orientation orders, and porosity on the heat transfer, as depicted in Figs. 4c, d and S16 [59, 60]. It is clear that the peak temperature of Ti_3_C_2_T_*x*_ fibers first rises and then decreases with an increase of Na_2_B_4_O_7_ mass fractions, reaching the highest temperature as well as the fastest heat transfer rate at 0.75 wt% Na_2_B_4_O_7_. In contrast, Ti_3_C_2_T_*x*_ fibers exhibit loosely arranged internal layers with more defects and greater air thermal resistance that hinders heat transfer as Na_2_B_4_O_7_ mass fraction is 0.25 wt%. The internal layers of Ti_3_C_2_T_*x*_ fibers with 0.75 wt% Na_2_B_4_O_7_ are tightly packed and well-oriented coupled with reduced ITR, which enhances physical contact and improves the alignment of the nanosheets, thereby significantly improving the heat transfer capabilities of the fibers as a result of the better construction of continuous and effective phonon transport pathways. However, when the Na_2_B_4_O_7_ content reaches 1.25 wt%, the low ITR between Ti_3_C_2_T_*x*_ nanosheets cannot compensate for the discontinuity in phonon transport pathways and the increased phonon scattering probability caused by the disorderly aggregation of nanosheets and the higher defect density within the fibers, ultimately leading to a reduction of heat transfer. The thermal conductivity coefficient (*λ*) of Ti_3_C_2_T_*x*_ fibers was measured using the cross-wire geometry method, where platinum heating wire served both as thermometers and heaters, and Ti_3_C_2_T_*x*_ fiber was mounted as test wire in a cross-geometry with the platinum heating wire [61, 62]. As shown in Fig. 4e, the *λ* of Ti_3_C_2_T_*x*_ fibers is 6.65 W m^−1^ K^−1^ with 0.25 wt% Na_2_B_4_O_7_, and it reaches its maximum value of 13 W m^−1^ K^−1^ when the Na_2_B_4_O_7_ content increases to 0.75 wt%. However, further increasing the Na_2_B_4_O_7_ concentration to 1.25 wt% results in a decrease of *λ* to 3.9 W m^−1^ K^−1^. The variation in *λ*, consistent with the trends from the finite element analysis, validates the impact of borate ester covalent bonding on the thermal conductivity of Ti_3_C_2_T_*x*_ fibers, where the borate ester bonds not only reduce ITR and improve heat transfer, but also drive the nanosheets into a more ordered and compactly stacked arrangement, thereby forming more efficient phonon transport pathways within the fibers and endowing Ti_3_C_2_T_*x*_ fibers with enhanced thermal conductivity (13 W m^−1^ K^−1^) (Fig. 4f).

### Joule Heating Performance of Ti_3_C_2_T_***x***_ Fibers

**Fig. 5:**
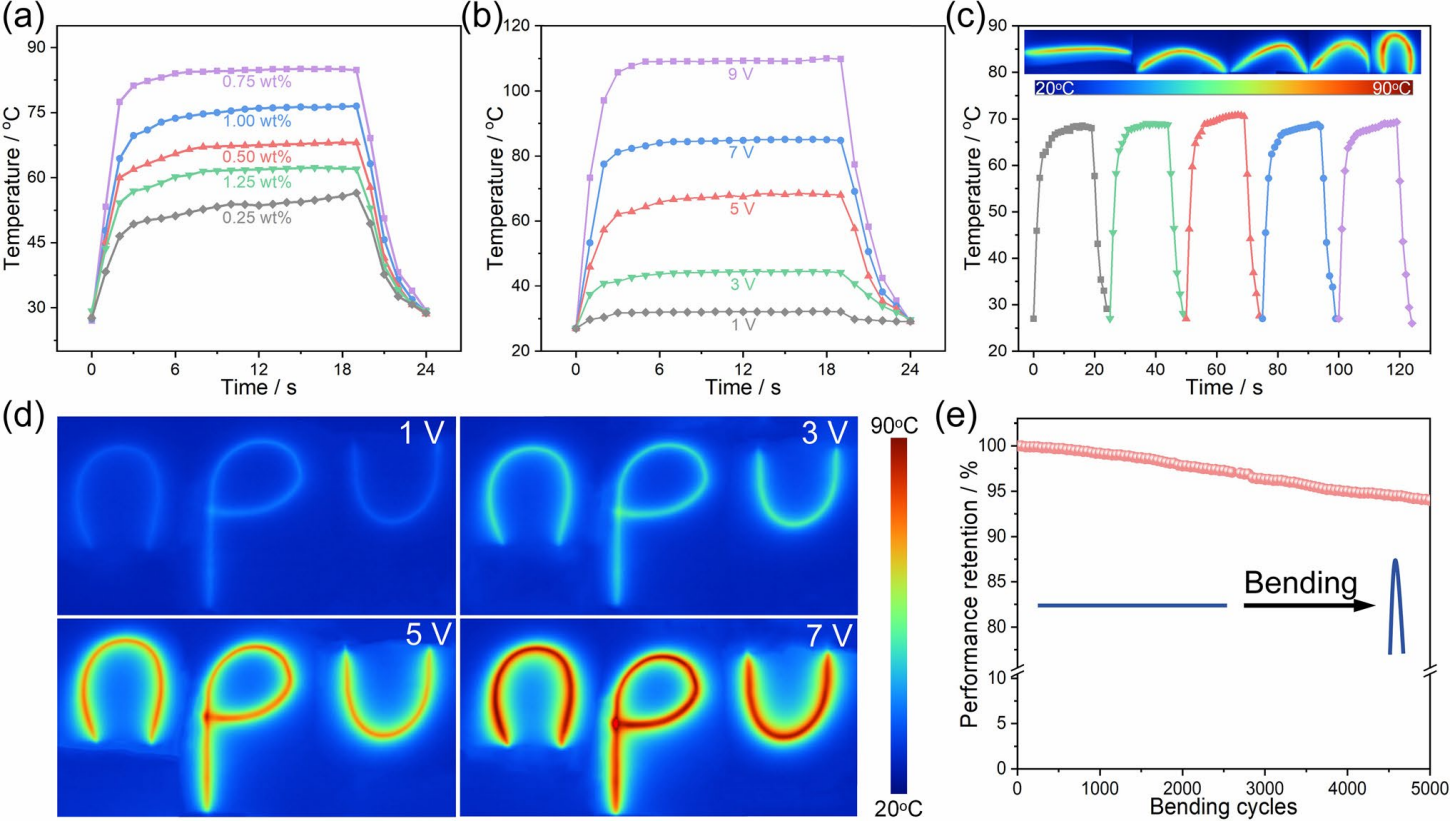
Joule heating performance of Ti_3_C_2_T_*x*_ fibers. **a** Temperature–time curves of single Ti_3_C_2_T_*x*_ fibers with different Na_2_B_4_O_7_ contents when applied DC voltage of 7 V. **b** Temperature–time curves of single Ti_3_C_2_T_*x*_ fibers with 0.75 wt% Na_2_B_4_O_7_ when applied DC voltage of 1 ~ 9 V. **c** Temperature–time curves and infrared thermal images of single Ti_3_C_2_T_*x*_ fibers at bending angles of 0° ~ 180°. **d** Infrared thermal images of Ti_3_C_2_T_*x*_ fibers with different letter shapes. **e** Temperature performance retention of Ti_3_C_2_T_*x*_ fibers after 5000 bending cycles

The Ti_3_C_2_T_*x*_ fibers obtained perform high thermal conductivity, mechanical strength, and electrical conductivity as well as superior Joule heating performance. Figure 5a presents the temperature–time curves for single Ti_3_C_2_T_*x*_ fibers with different mass fractions of Na_2_B_4_O_7_. It is apparent that all Ti_3_C_2_T_*x*_ fibers immediately generate heat, with surface temperatures rapidly increasing and reaching a steady state around 3 s when applying the working DC voltage of 7 V, indicating a rapid Joule heating response. Notably, Ti_3_C_2_T_*x*_ fibers achieve the highest equilibrium temperature of 85.1°C with 0.75 wt% Na_2_B_4_O_7_. The equilibrium temperatures of Ti_3_C_2_T_*x*_ fibers can be finely tuned by applying varying DC voltages from 1 to 9 V, with temperatures rising from 32.1 to 109.3 °C when the content of Na_2_B_4_O_7_ is 0.75 wt%, demonstrating their adaptable and tunable Joule heating performance (Fig. 5b). Moreover, the mechanical stability of Ti_3_C_2_T_*x*_ fibers needs to be considered for further practical applications in thermal management [63, 64]. As shown in Fig. 5c, the mechanical stability of Ti_3_C_2_T_*x*_ fibers was confirmed by the results that the temperature–time curves of Ti_3_C_2_T_*x*_ fibers at bending angles from 0° to 180° under a 5 V applied voltage remain consistent across different bending angles with an evenly distributed temperature according to the infrared thermal images. Figure 5d displays infrared thermal images of Ti_3_C_2_T_*x*_ fibers shaped into the letters (npu) under applied voltage, where each letter was formed from fibers of the same length. It is observed that the equilibrium temperatures of the differently shaped letters increase with the applied voltage, exhibiting uniform and consistent temperature distribution. This also proves the excellent flexibility and reliable Joule heating performance of the Ti_3_C_2_T_*x*_ fibers. Additionally, single Ti_3_C_2_T_*x*_ fibers exhibit outstanding cyclic durability, maintaining the performance retention of ~ 94% after 5000 bending cycles (Fig. 5e). These results demonstrate the exceptional and stable Joule heating performance of Ti_3_C_2_T_*x*_ fibers, highlighting their significant potential in wearable thermal management applications.

## Conclusions

This work presented a simple and efficient interface covalent crosslinking enhancement strategy, where strong covalent interactions between borate and the hydroxyl groups on the Ti_3_C_2_T_*x*_ surface enabled the successful assembly of Ti_3_C_2_T_*x*_ nanosheets into continuous Ti_3_C_2_T_*x*_ fibers via wet spinning. DFT calculations and experimental studies showed that the formation of trace and optimal amounts of borate ester covalent bonding significantly enhanced the interlayer interactions within Ti_3_C_2_T_*x*_ fibers, while dramatically reducing interlayer porosity and promoting sheet alignment, resulting in Ti_3_C_2_T_*x*_ fibers with outstanding mechanical and electrical properties. When the Na_2_B_4_O_7_ content is 0.75 wt%, Ti_3_C_2_T_*x*_ fibers exhibit optimal tensile strength of 188.72 MPa and Young’s modulus of 52.42 GPa as well as electrical conductivity of 7781 S cm^−1^. More importantly, EMD simulations and finite element analysis demonstrated that the formation of borate ester covalent bonds reduced the ITR between Ti_3_C_2_T_*x*_ nanosheets, while the low ITR, combined with high alignment and densification of nanosheets, significantly boosted the thermal conductivity of Ti_3_C_2_T_*x*_ fibers, as confirmed by cross-wire geometry method, which achieved optimal *λ* of 13 W m^−1^ K^−1^ with 0.75 wt% Na_2_B_4_O_7_. Additionally, the excellent Joule heating performance of these fibers was displayed. The results reveal that the demonstrated strategy opens up new avenues for the application of Ti_3_C_2_T_*x*_ in the development of fibers with high electrical conductivity and thermal conductivity for smart textiles. Indeed, a great number of multifunctional fibers can be generally assembled through such an effective strategy from various nanomaterials to meet diverse requirements.

## Supplementary Information

Below is the link to the electronic supplementary material.

Supplementary file1 (DOCX 4369 KB)

Supplementary file2 (MP4 7737 KB)
