# Supplementary material for: Thermally Conductive Ti3C2Tx Fibers with Superior Electrical Conductivity
Source: Nanomicro Lett. 2025 Apr 27;17:235. doi: 10.1007/s40820-025-01752-x (PMC12034612; doi:10.1007/s40820-025-01752-x)
Supplement: Supplementary file 1 — Supplementary file1 (DOCX 4369 KB) [file 40820_2025_1752_MOESM1_ESM.docx]

Supporting Information for

**Thermally Conductive Ti_3_C_2_T*_x_* Fibers with Superior Electrical Conductivity**

Yuxiao Zhou^1^, Yali Zhang^1^*, Yuheng Pang^1^, Hua Guo^1^, Yongqiang Guo^1^, Mukun Li^1^, Xuetao Shi^1^, Junwei Gu^1^*

^1^Shaanxi Key Laboratory of Macromolecular Science and Technology, School of Chemistry and Chemical Engineering, Northwestern Polytechnical University, Xi’an, Shaanxi 710072, P. R. China

*Corresponding authors. E-mail: [yalizhang@nwpu.edu.cn](mailto:yalizhang@nwpu.edu.cn) (Yali Zhang), [gjw@nwpu.edu.cn](mailto:gjw@nwpu.edu.cn) (Junwei Gu)

**S1** **Preparation of Ti_3_C_2_T*_x_* dispersion**

Firstly, 1.6 g LiF was added to 20 mL of 9M HCl and stirred for 30 min until completely dissolved. 1.0 g of Ti_3_ACl_2_ powder was slowly added and stirred at 50 ^o^C for 48 h under argon atmosphere. After the reaction, the mixture was washed about 8 cycles repeatedly with deionized water. Each cycle involved 5 min of centrifugation at 3500 r/min. Until the supernatant solution reached pH≈7, the resulting sediment was dispersed in deionized water with continuously vibration for 20 min. Next, the solution was then centrifuged at 1500 r/min for 30 min, and the supernatant solution was then taken and centrifuged at 4500 r/min for 20 min. Finally, the sediments were dispersed into deionized water to obtain Ti_3_C_2_T*_x_* dispersion of various concentrations.

**S2 Characterization**

Fourier transform infrared spectroscopy (FT-IR, Bruker Tensor II, Bruker, Germany) was used to characterize the functional groups of Ti_3_C_2_T*_x_* nanosheets and Ti_3_C_2_T*_x_* fibers. X-ray photoelectron spectroscopy (XPS, Kratos Axis Ultra DLD, UK) was used to analyze the elemental composition and binding energy variations of Ti_3_C_2_T*_x_* nanosheets and Ti_3_C_2_T*_x_* fibers. X-ray diffraction (XRD) spectra were obtained to identify the crystal phase of Ti_3_C_2_T*_x_* nanosheets and Ti_3_C_2_T*_x_* fibers by an X-ray diffractometer (D8 Advance, Bruker, Germany) equipped with a Cu K𝛼 target radiation source (𝜆 = 1.54 Å). The dimensions of Ti_3_C_2_T*_x_* nanosheets and the morphologies of Ti_3_C_2_T*_x_* fibers were characterized using scanning electron microscopy (SEM, Verios G4, FEI, USA). The topography of Ti_3_C_2_T*_x_* nanosheets was observed *via* atomic force microscope (AFM, Dimension Icon, Bruker, Germany) in tapping mode. The morphology and crystal structure of Ti_3_C_2_T*_x_* nanosheets were taken by transmission electron microscope (TEM, Talos F200X, FEI, USA). The optical birefringence of Ti_3_C_2_T*_x_* dispersion was recorded under a polarized optical microscope (POM, DM4P, Leica, Germany). The rheological properties of Ti_3_C_2_T*_x_* dispersion were obtained using a rheometer (DHR-20, ‌TA Instruments, USA) under both steady shear and dynamic oscillatory conditions. The viscoelastic properties of Ti_3_C_2_T*_x_* dispersion were investigated by measuring the storage and loss modulus as a function of frequency from 0.01 to 100 Hz. The strain amplitude remained at 0.1% with a gap of 1 mm at 25 ^o^C for the frequency sweep. The wide-angle X-ray scattering (WAXS) and small-angle X-ray scattering (SAXS) patterns of Ti_3_C_2_T*_x_* fibers were conducted on a SAXS/WAXS system (XEUSS 3.0, Xenocs SAS, France) utilizing a Cu-K𝛼 targeted radiation source with a beam diameter of 900 nm on SAXS/WAXS (500 K) detectors. The electrical conductivity of Ti_3_C_2_T*_x_* fibers was tested using a multimeter (Keithley 2700, Keithley Instruments, USA) *via* a standard two-probe method. The mechanical properties of Ti_3_C_2_T*_x_* fibers were evaluated using a universal tensile testing instrument (XQ-1C, Shanghai New Fiber Instrument, China) at a loading rate of 1 mm/min with a 10 N sensor at room temperature. Infrared thermal images of Ti_3_C_2_T*_x_* fibers were captured using infrared thermography (Ti 300, Fluke Co., USA). The area of Ti_3_C_2_T*_x_* fibers was measured by SEM. The thermal conductivity (*λ*) of Ti_3_C_2_T*_x_* fibers was measured using a cross-wire geometry.

**S3** ***λ* of Ti_3_C_2_T*_x_* fibers measured using a cross-wire geometry**

The Platinum wire with a diameter of 10 μm and a length of about 6 mm was used as the heating wire, and the heating wire and the test wire (Ti_3_C_2_T*_x_* fiber) were welded to the copper leads as thermostatic heat sinks. The heating wire and the fiber were mounted in a cross geometry, as shown in **Fig. S1**, Supporting Information. In this geometry, the heating wire was perpendicular to an external magnetic field, while the fiber was parallel to the field. The self-made vacuum chamber was continuously evacuated by a molecular pump to maintain a high vacuum level of 10^-4^ Pa so the effect of convective heat transfer was ignored. The four-point probe technique was adopted. In the DC measurement, the constant current source (Agilent B2901A) was used to apply different currents to the heating wire, and the digital multimeter (Agilent 3458A) was used to measure the voltage across the heating wire. During the AC measurement, the lock-in amplifier (SR7265) was used to output AC signals with different frequencies of 0.1-1 Hz, and the 3*ω* voltage across the heating wire was measured by the same lock-in amplifier. To control the contact between the heating wire and the fiber, a direct current was imposed to the heating wire and a magnetic field was applied perpendicular to the current. The deflection of the heating wire could be in situ changed under different Ampère forces so as to contact and separate the fiber. When the two wires contacted each other, the temperature rise in the heating wire decreased since some heat would conduct out through the fiber. It was recognized that the thermal contact resistance of the bare junction plays a significant role in determining the thermal conductivity of the fiber. To overcome this problem, an AC was fed into the heating wire to measure the thermal contact resistance. *λ* of the fiber was obtained by comparing the temperature change of the heating wire before and after lapping the fiber.

**S4 DFT calculations of** **borate ester bonded covalently Ti_3_C_2_T*_x_* nanosheets**

The density functional theory (DFT) calculations were undertaken using the CP2K software package. Employing the projector augmented wave (PAW) method. The Perdew-Burke-Ernzerhof (PBE) exchange-correlation energy was utilized within the generalized gradient approximation (GGA). A plane-wave basis with an energy cutoff of 400 eV was employed. A 4 × 4 × 1 k-point mesh was used for sampling the Brillouin zone with the gamma-centered method. The DFT-D3 correction was applied to account for van der Waals interactions. A vacuum thickness of no less than 15 Å in the Z direction was used to eliminate interactions between periodic cells. Convergence thresholds for forces and energies were set to 10^-6^ eV and 0.02 eV/Å, respectively. The charge density difference (CDD) and electron localization function (ELF) were performed by Multiwfn 3.8(dev) software packages.

**S5 EMD simulation of borate ester bonded covalently Ti_3_C_2_T_x_ nanosheets**

Equilibrium molecular dynamics (EMD) simulation based on the Green-Kubo method was performed using large-scale atomic/molecular massively parallel simulator (LAMMPS). A face-centered cubic cell of Ti_3_C_2_T*_x_* was constructed, and then the cell was expanded to tune the atoms and doped with different ratios of B elements to build different models. The box over sizes were all set to 56 Å × 50 Å × 30 Å. All simulations were performed in the universal force field (UFF), during which the time step was set to 0.01 fs and the initial velocity of the atoms was randomly sampled from a Gaussian distribution. The conjugate gradient method in LAMMPS was used to minimize the energy and relax 5 ns at 300 K in the NPT ensemble, followed by another 5 ns in the NVT ensemble. Finally, the interfacial thermal resistance (ITR) between Ti_3_C_2_T*_x_* nanosheets with different amounts of B in the 300 K NVE ensemble was calculated by EMD simulation where there was basically no change inside the material when the heat current auto correlation function, temperature and energy tended to be stable. By calculating the change of internal heat flow from disequilibrium to equilibrium, the *λ*, and thus ITR between Ti_3_C_2_T*_x_* nanosheets was obtained.

Based on the Green-Kubo linear-response theory, the autocorrelation heat flow was obtained by counting the potential energy, kinetic energy and stress tensor of each particle in the system, and the ITR between Ti_3_C_2_T*_x_* nanosheets was obtained from the Green-Kubo equation and the thermal resistance calculation formula, which were given by the following equations:

$\lambda=\frac{1}{3Vk_{B}T_{2}}\int_{0}^{\infty} \left[ J\left( t \right) \right]*\left[ J\left( 0 \right) \right]dt$ (S1)

where *k*_B_ was the Boltzmann constant, *V* was the volume of the system, and *J* was the effective heat flow, *λ* was the thermal conductivity of the adjacent nanosheets.

$J\left( t \right)=\frac{d}{dt}\sum_{i} r_{i}\left( t \right)e_{i}\left( t \right)$ (S2)

where *i* denoted the spatial position of particle *i* and *e_i_* denoted the sum of potential and kinetic energies of particle *i*.

$e_{i}=\frac{1}{2}mv_{i}+\frac{1}{2}\sum\varphi\left( r_{ij} \right)$ (S3)

where *i* and *j* denoted different particles, *v* was the velocity of particle *i*, and (*ij*) is the interatomic potential energy of *i* and *j*.

$R=\frac{A}{\lambda L}$ (S4)

where *R* was the ITR, *L* was the thickness and *A* was the cross-sectional area of the adjacent nanosheets.

**S6 Finite element analysis of Ti_3_C_2_T*_x_* fibers**

Finite element analysis was used to simulate the transient thermal response of Ti_3_C_2_T*_x_* fibers with different mass fractions of borate during heating with finite element analysis software COMSOL Multiphysics 6.2. The module was solid heat transfer. The domain-specific simulation models of all Ti_3_C_2_T*_x_* fibers with different mass fractions of borate were set to 50 μm × 100 μm. The grid generation was described as using the extremely fine grid division method. Parameters including ITR of borate ester bonded covalently Ti_3_C_2_T*_x_* nanosheets simulated by EMD in S2, interlayer spacing (d-spacing), orientation orders and porosity were used for finite element simulation. The initial conditions of the five simulation models were set to 25 ^o^C, and then linear heat sources with a temperature of 50 ^o^C were set at the bottom of the models, respectively, to measure the internal heat transfer of different models under the transient state (10 ns).

**Supplementary Figures**


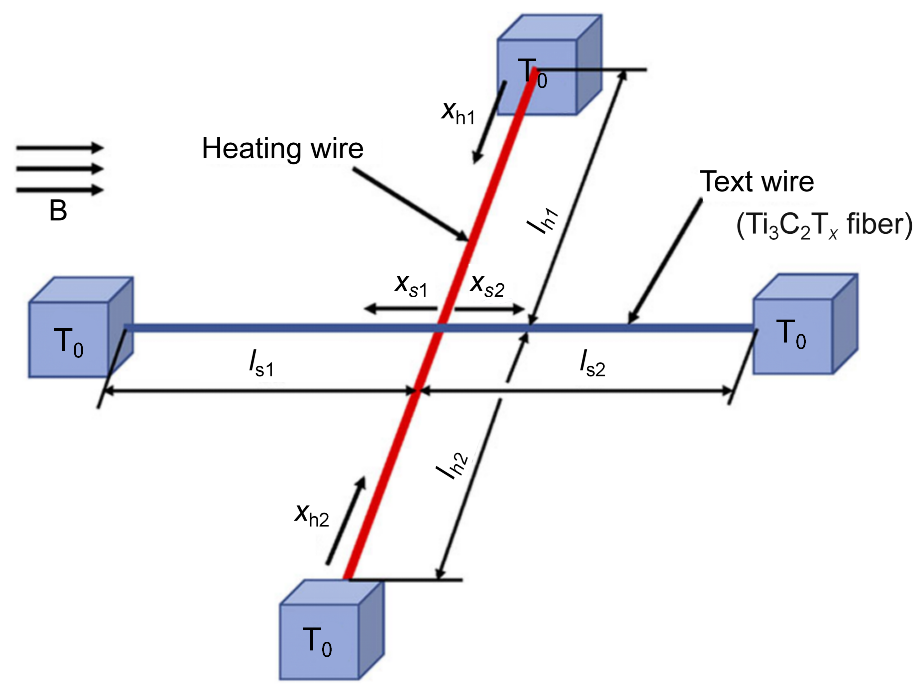


**Fig. S1** Principle of the cross-wire geometry method for measuring the *λ* of Ti_3_C_2_T*_x_* fiber


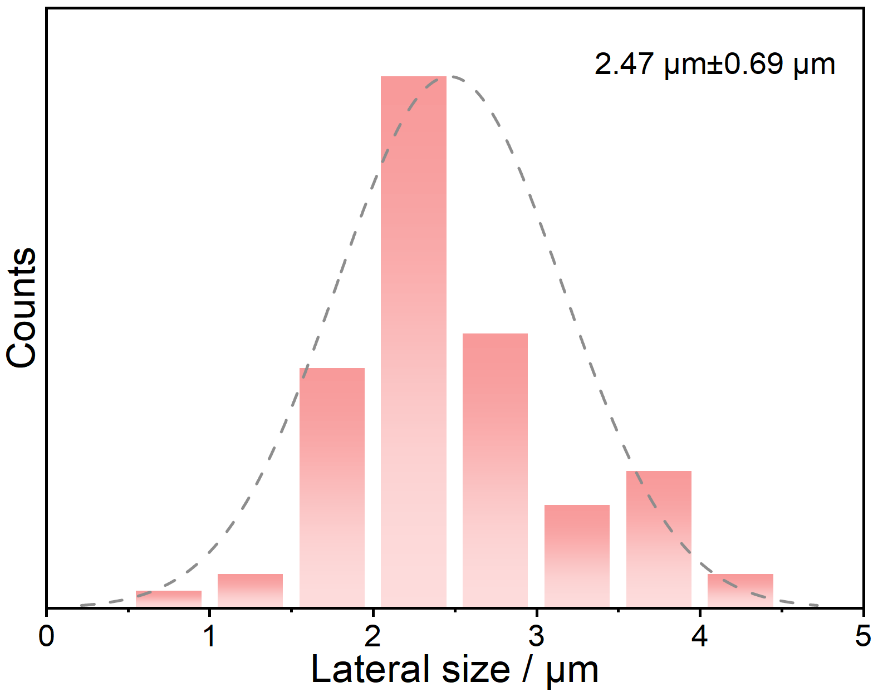


**Fig. S2** Size distribution of Ti_3_C_2_T*_x_* nanosheets


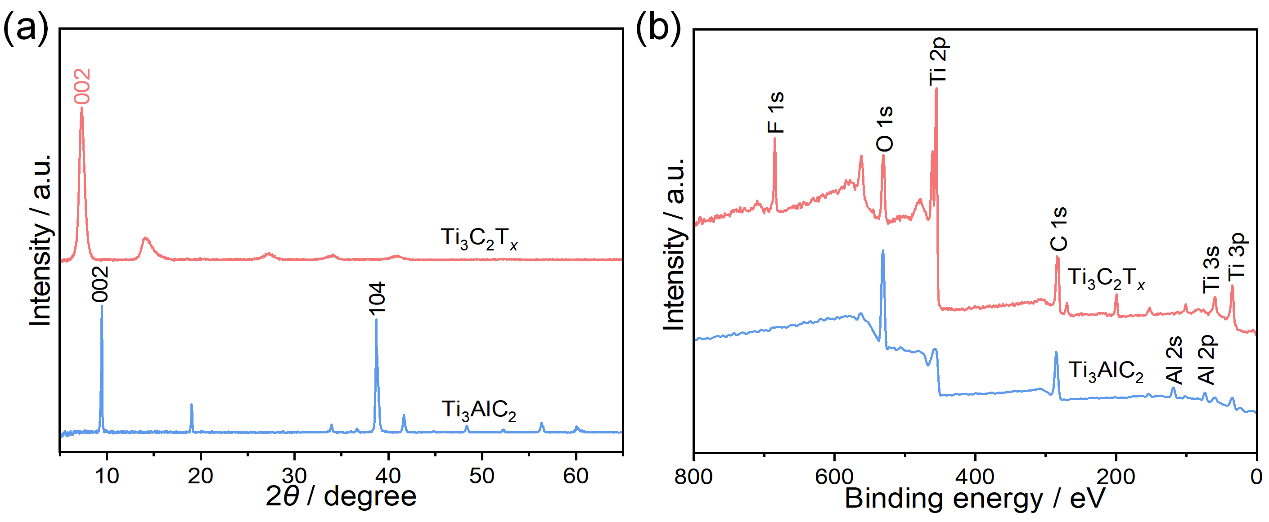


**Fig. S3** (**a**) XRD and (**b**) XPS spectra of Ti_3_AlC_2_ and Ti_3_C_2_T*_x_*


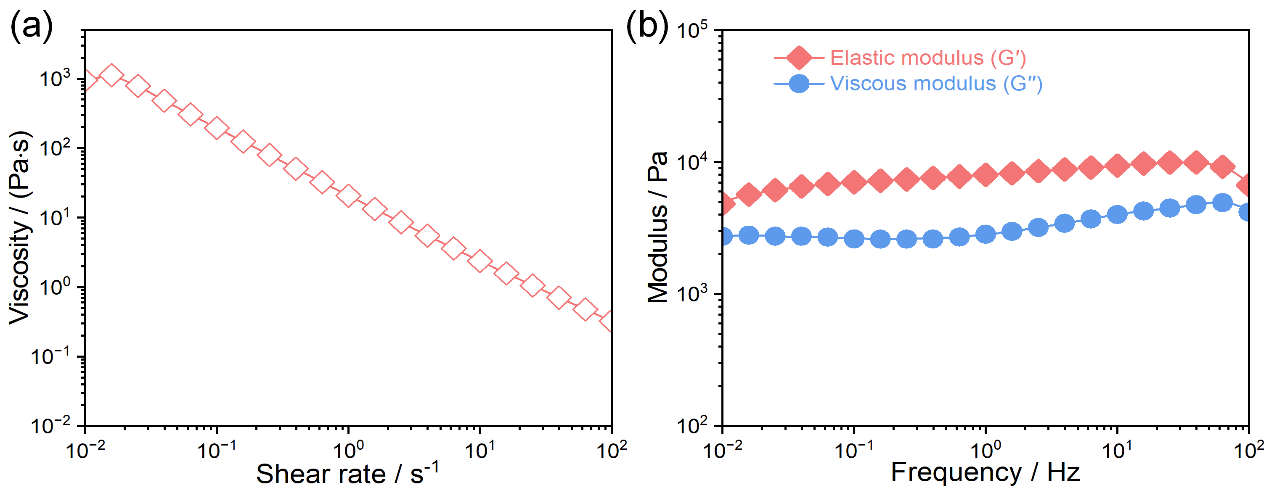


**Fig. S4** (**a**) Viscosity as a function of shear rate versus shear rate. (**b**) Storage and loss modulus as a function of frequency of Ti_3_C_2_T*_x_* liquid-crystalline dispersion at a concentration of 25 mg/mL


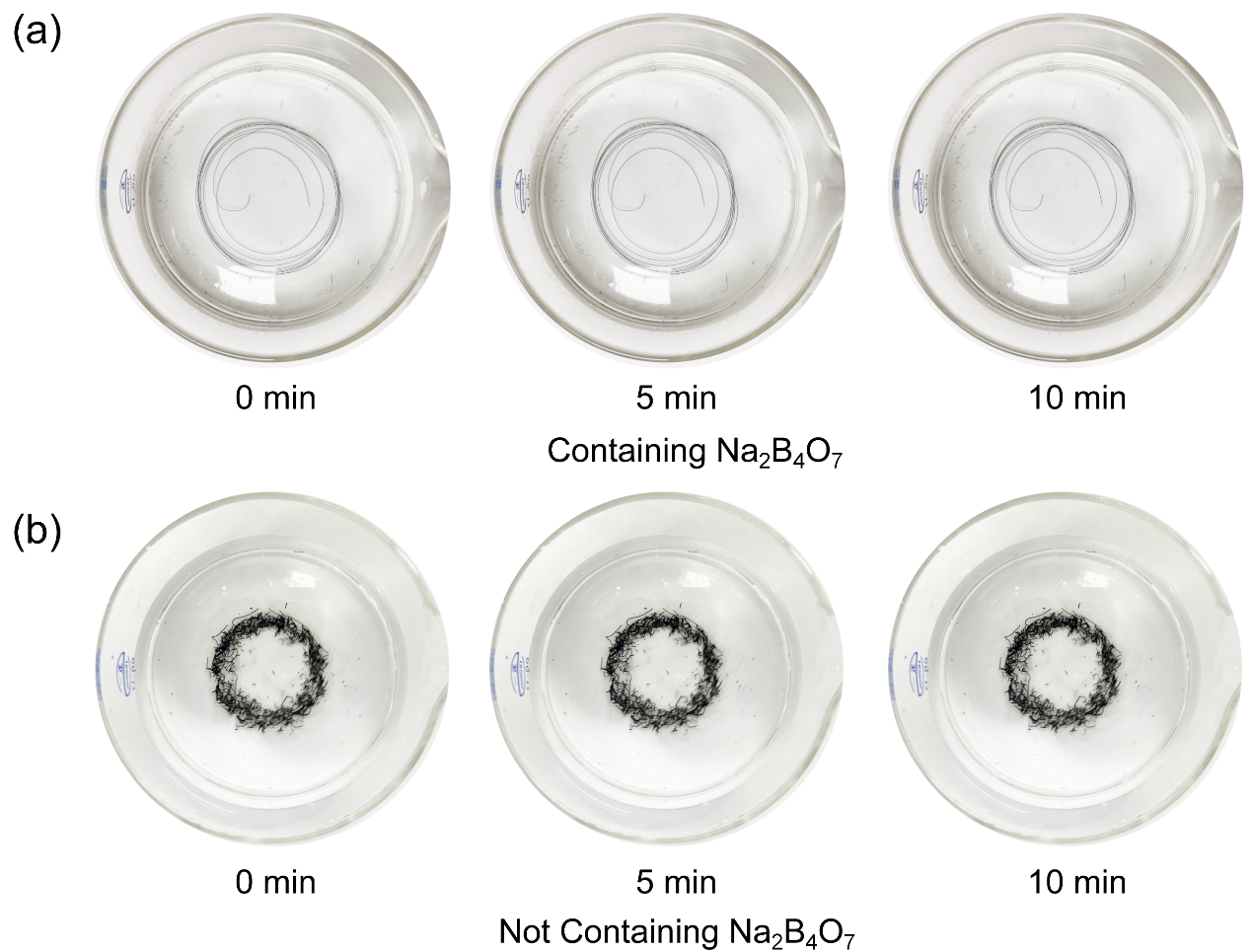


**Fig. S5** Photographs of (**a**) the formation of continuous Ti_3_C_2_T*_x_* gel fibers in a coagulation bath containing Na_2_B_4_O_7_ and (**b**) the failure to form continuous Ti_3_C_2_T*_x_* gel fibers in a coagulation bath without Na_2_B_4_O_7_


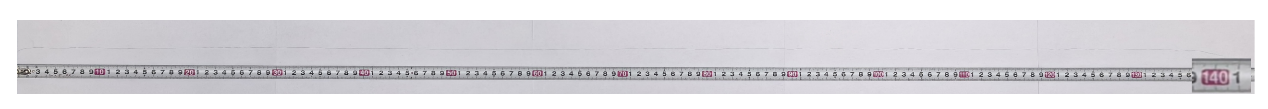


**Fig. S6** Photograph of continuous Ti_3_C_2_T*_x_* fiber over 1 m (1.4 m)


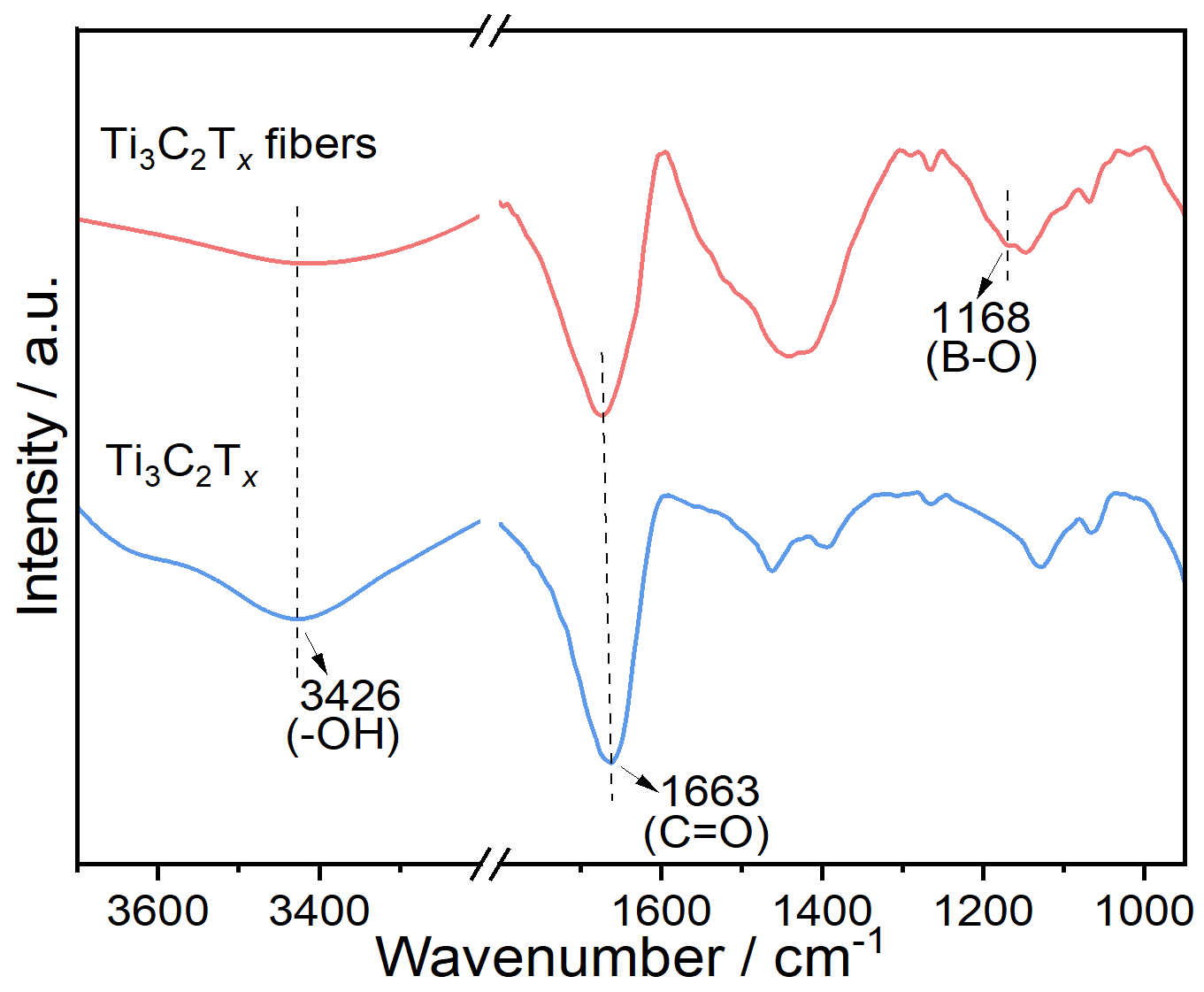


**Fig. S7** FT-IR spectra of Ti_3_C_2_T*_x_* and Ti_3_C_2_T*_x_* fibers


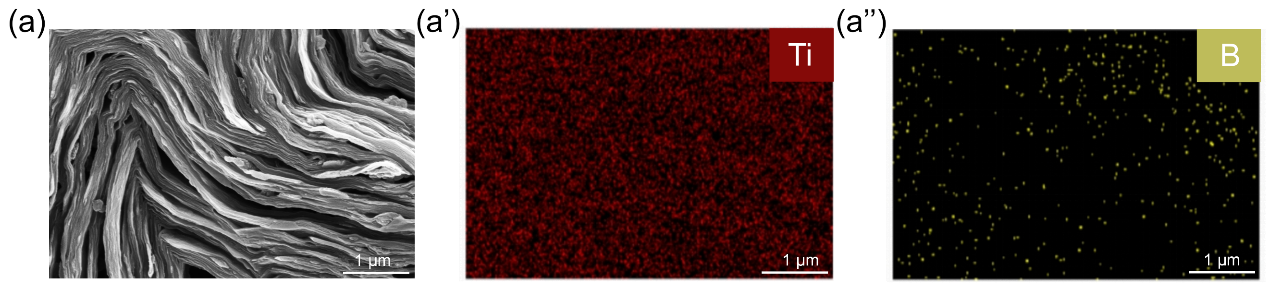


**Fig. S8** (**a**) SEM image of the cross-section for Ti_3_C_2_T*_x_* fiber and corresponding energy dispersive spectrometer (EDS) element distribution of (**a’**) Ti and (**a”**) B


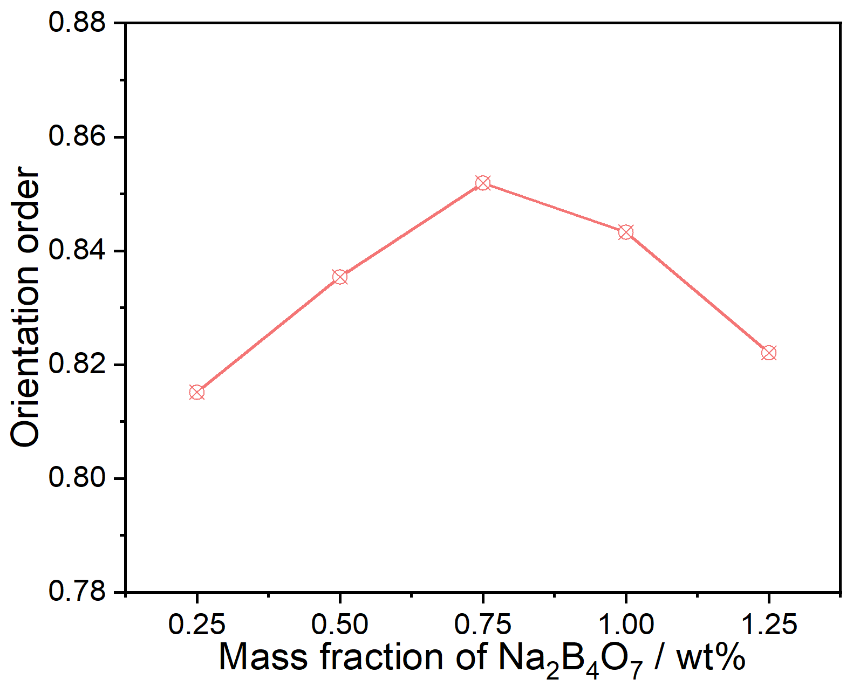


**Fig. S9** Orientation order of Ti_3_C_2_T*_x_* fibers with different Na_2_B_4_O_7_ contents


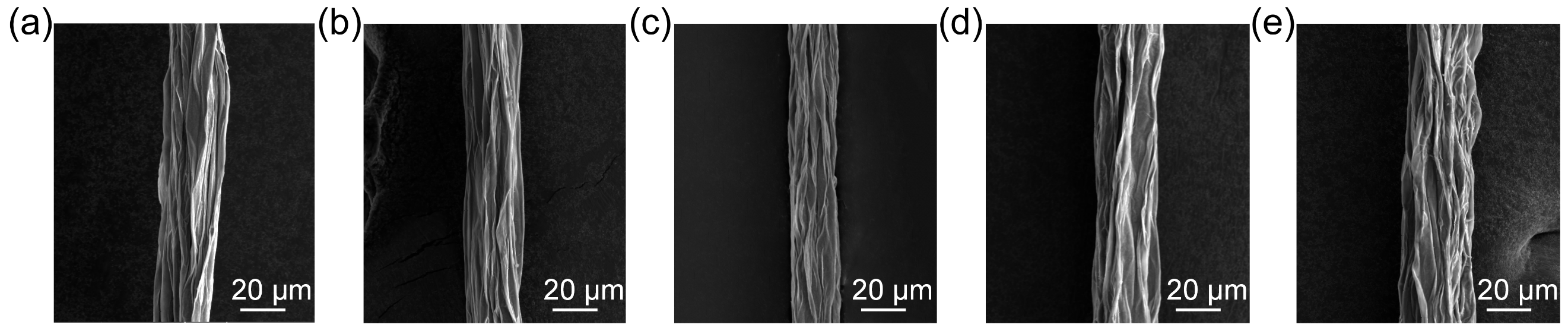


**Fig. S10** SEM images of the side section for Ti_3_C_2_T*_x_* fibers with Na_2_B_4_O_7_ content from (**a**) 0.25 wt%, (**b**) 0.50 wt%, (**c**) 0.75 wt%, (**d**) 1.00 wt% and (**e**) 1.25 wt%


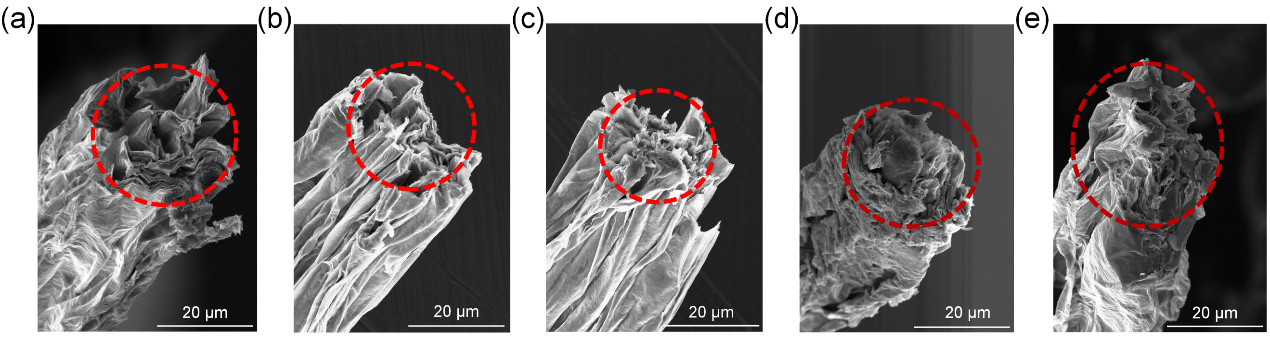


**Fig. S11** SEM images of the cross section for Ti_3_C_2_T*_x_* fibers with Na_2_B_4_O_7_ content from (**a**) 0.25 wt%, (**b**) 0.50 wt%, (**c**) 0.75 wt%, (**d**) 1.00 wt% and (**e**) 1.25 wt%


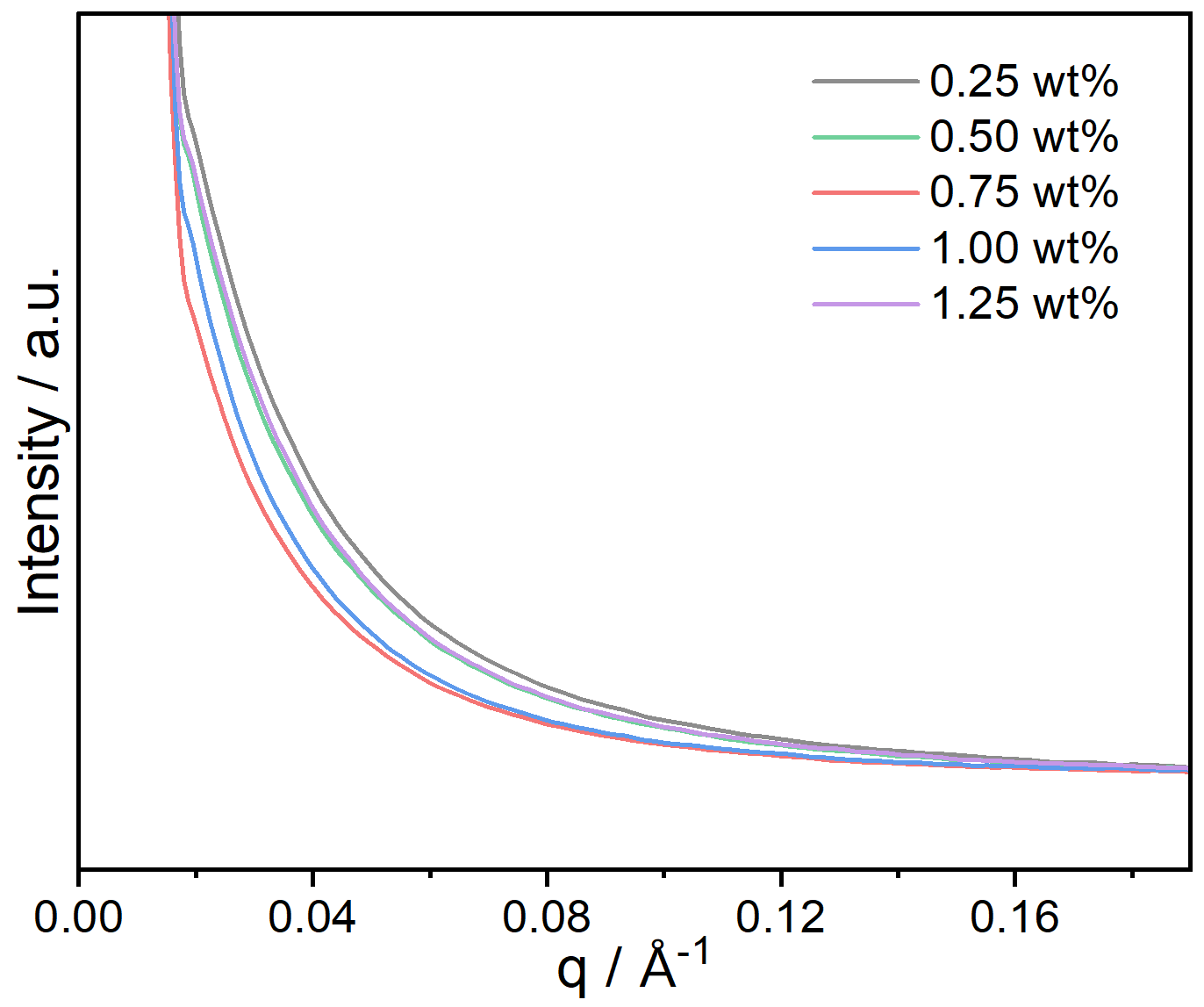


**Fig. S12** Plots of the intensity *vs.* q for Ti_3_C_2_T*_x_* fibers with different Na_2_B_4_O_7_ contents according to the SAXS patterns


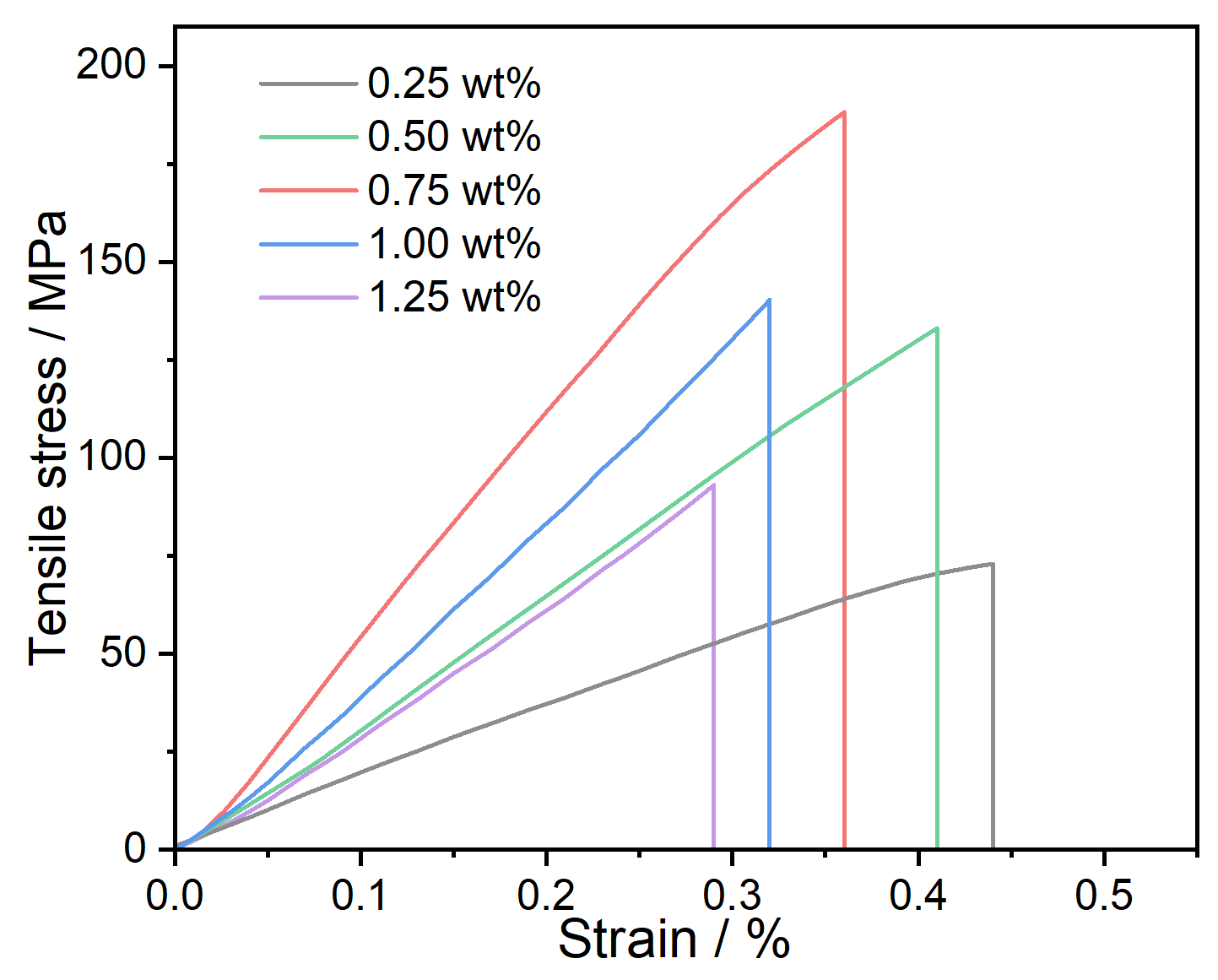


**Fig. S13** Stress-strain curves of Ti_3_C_2_T*_x_* fibers with different Na_2_B_4_O_7_ contents


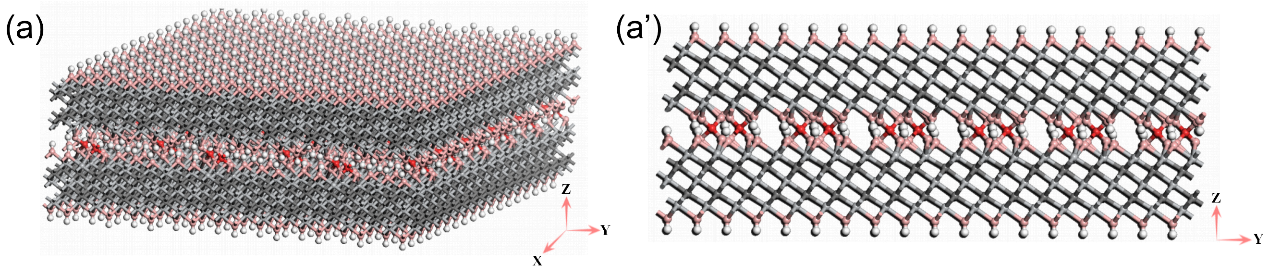


**Fig. S14** (**a**) Three-dimensional structure and (**a’**) cross-sectional view of Ti_3_C_2_T*_x_* nanosheets bonded by covalent borate ester


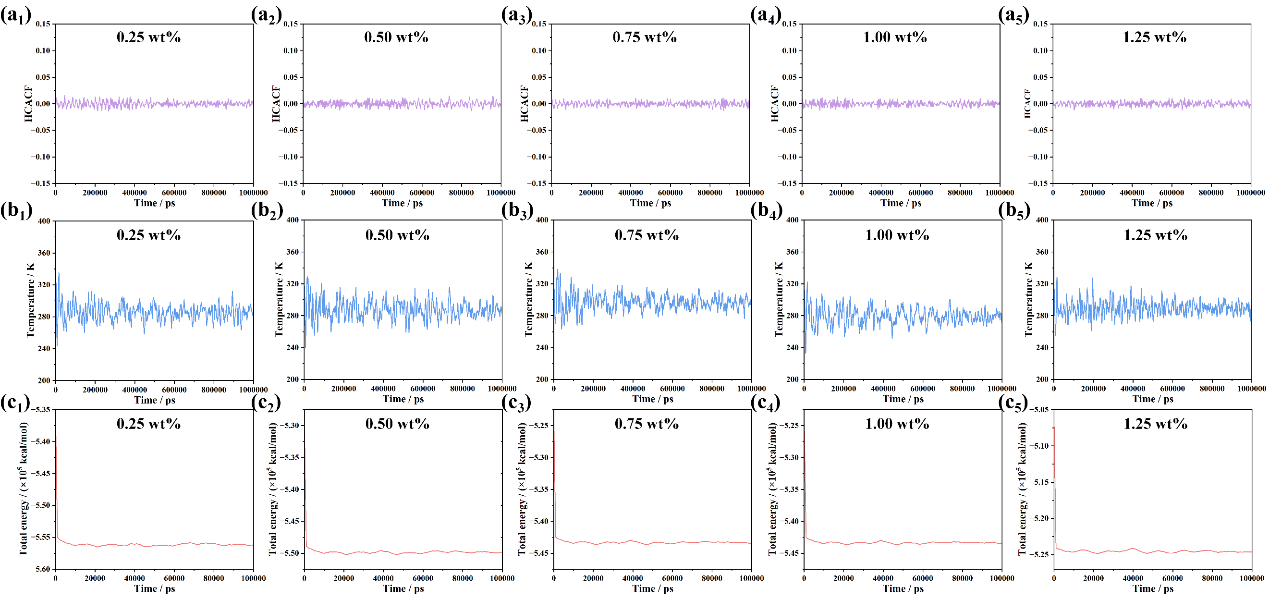


**Fig. S15** Curves of (**a_1_-a_5_**) the heat current auto correlation function, (**b_1_-b_5_**) temperature and (**c_1_-c_5_**) total energy for borate ester covalently bonded Ti_3_C_2_T*_x_* nanosheets with Na_2_B_4_O_7_ contents from 0.25 wt%, 0.50 wt%, 0.75 wt%, 1.00 wt% and 1.25 wt%


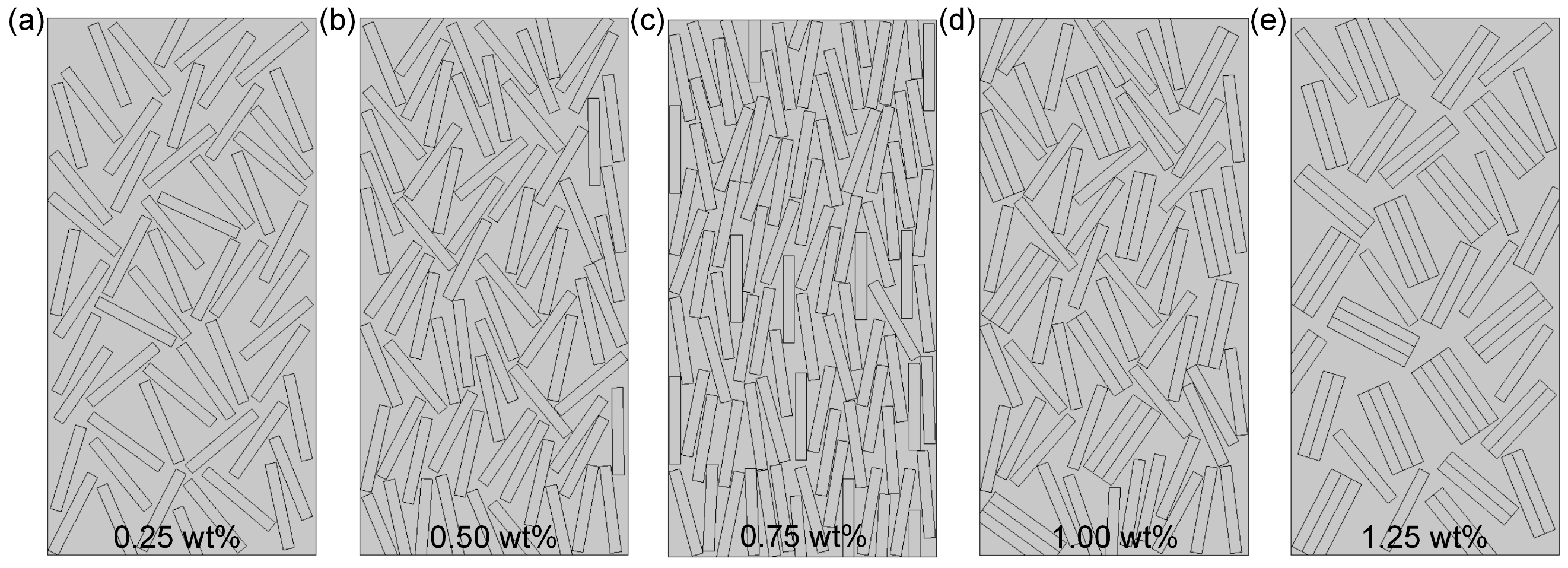


**Fig. S16** FEA models of Ti_3_C_2_T*_x_* fibers (where the long strips are Ti_3_C_2_T*_x_* nanosheets) with Na_2_B_4_O_7_ contents from (**a**) 0.25 wt%, (**b**) 0.50 wt%, (**c**) 0.75 wt%, (**d**) 1.00 wt% and (**e**) 1.25 wt%

**Table S1** B content in Ti_3_C_2_T*_x_* fibers prepared with different Na_2_B_4_O_7_ contents, determined by XPS characterizations

| Na_2_B_4_O_7_ content in coagulant used for the fabrication of Ti_3_C_2_T*_x_* fibers (wt%) | B content in Ti_3_C_2_T*_x_* fibers (wt%) |
| --- | --- |
| 0.25 | 0.052 |
| 0.50 | 0.098 |
| 0.75 | 0.142 |
| 1.00 | 0.193 |
| 1.25 | 0.246 |

**Table S2** Comparisons of electrical conductivity and tensile strength of Ti_3_C_2_T*_x_* fibers prepared with 0.75 wt% Na_2_B_4_O_7_ against other reported Ti_3_C_2_T*_x_*-based fibers and Ti_3_C_2_T*_x_* fibers

| Number | Fiber material | Tensile strength  (MPa) | Conductivity  (S/cm) | Refs. |
| --- | --- | --- | --- | --- |
| 1 | Ti_3_C_2_T*_x_*/CNT composite fiber | 38.4 | 2.7 | [S1] |
| 2 | Ti_3_C_2_T*_x_*/rGO composite fiber | 145 | 28 | [S2 |
| 3 | Ti_3_C_2_T*_x_*/rGO composite fiber | 110.7 | 743.1 | [S3] |
| 4 | Ti_3_C_2_T*_x_*/CDs composite fiber | 80 | 1201 | [S4] |
| 5 | Ti_3_C_2_T*_x_*/CNT composite fiber | 161 | 1715 | [S5] |
| 6 | Ti_3_C_2_T*_x_*/PU composite fiber | 37 | 1195 | [S6] |
| 7 | Ti_3_C_2_T*_x_*/CNF composite fiber | 46 | 916 | [S7] |
| 8 | Ti_3_C_2_T*_x_*/PEDOT:PSS composite fiber | 58.1 | 1489 | [S8] |
| 9 | MXene/CMC composite fiber | 81 | 1073 | [S9] |
| 10 | MP/Ag/MP composite fiber | 82.7 | 1575 | [S10] |
| 11 | Ti_3_C_2_T*_x_*/Aramid fiber | 104 | 1025 | [S11] |
| 12 | Ti_3_C_2_T*_x_*/ANF composite fiber | 130 | 25.15 | [S12] |
| 13 | Ti_3_C_2_T*_x_*/CNC composite fiber | 60 | 3000 | [S13] |
| 14 | Ti_3_C_2_T*_x_* fiber | 36 | 1801 | [S9] |
| 15 | Ti_3_C_2_T*_x_* ribbon fiber | 40 | 2548 | [S14] |
| 16 | *N*-round Ti_3_C_2_T*_x_* fiber | 52 | 1260 | [S15] |
| 17 | *N*-flat Ti_3_C_2_T*_x_* fiber | 60 | 2680 | [S15] |
| 18 | Zn-Ti_3_C_2_T*_x_* fiber | 150.7 | 3637.9 | [S16] |
| 19 | Ti_3_C_2_T*_x_* fiber | 40.5 | 7748 | [S17] |
| 20 | Ti_3_C_2_T*_x_* fiber | 63.9 | 7713 | [S18] |
| 21 | *O*-flat Ti_3_C_2_T*_x_* fiber | 118 | 7200 | [S15] |
| 22 | Ti_3_C_2_T*_x_* fiber | 188.7 | 7781 | This work |

**Supplementary References**

1. C.Y. Yu, Y.J. Gong, R.Y. Chen, M.Y. Zhang, J.Y. Zhou et al., A solid-state fibriform supercapacitor boosted by host–guest hybridization between the carbon nanotube scaffold and MXene nanosheets. Small **14**(29), 1801203 (2018). <https://doi.org/10.1002/smll.201801203>
2. Q. Yang, Z. Xu, B. Fang, T. Huang, S. Cai et al., MXene/graphene hybrid fibers for high performance flexible supercapacitors. J. Mater. Chem. A **5**(42), 22113–22119 (2017). <https://doi.org/10.1039/c7ta07999k>
3. N. He, S. Patil, J. Qu, J. Liao, F. Zhao et al., Effects of electrolyte mediation and MXene size in fiber-shaped supercapacitors. ACS Appl. Energy Mater. **3**(3), 2949–2958 (2020). <https://doi.org/10.1021/acsaem.0c00024>
4. H. Wang, W. Zhao, Z. Zhang, W. Hou, L. Yin et al., Synergizing electron and ion transports of Ti_3_C_2_T_X_ MXene fiber *via* dot-sheet heterostructure and covalent Ti─C─Ti cross-linking for efficient charge storage and thermal management. Adv. Funct. Mater. **34**(48), 2408508 (2024). <https://doi.org/10.1002/adfm.202408508>
5. X. Zhao, J. Zhang, K. Lv, N. Kong, Y. Shao et al., Carbon nanotubes boosts the toughness and conductivity of wet-spun MXene fibers for fiber-shaped super capacitors. Carbon **200**, 38–46 (2022). <https://doi.org/10.1016/j.carbon.2022.08.045>
6. X. Zhao, J. Zhang, K. Lv, N. Kong, Y. Shao et al., Carbon nanotubes boosts the toughness and conductivity of wet-spun MXene fibers for fiber-shaped super capacitors. Carbon **200**, 38–46 (2022). <https://doi.org/10.1016/j.carbon.2022.08.045>
7. Q. Liang, K. Liu, T. Xu, Y. Wang, M. Zhang et al., Interfacial modulation of Ti3C2Tx MXene by cellulose nanofibrils to construct hybrid fibers with high volumetric specific capacitance. Small **20**(17), 2307344 (2024). <https://doi.org/10.1002/smll.202307344>
8. J. Zhang, S. Seyedin, S. Qin, Z. Wang, S. Moradi et al., Highly conductive Ti_3_C_2_T_x_ MXene hybrid fibers for flexible and elastic fiber-shaped supercapacitors. Small **15**(8), 1804732 (2019). <https://doi.org/10.1002/smll.201804732>
9. H. Wang, Y. Wang, J. Chang, J. Yang, H. Dai et al., Nacre-inspired strong MXene/cellulose fiber with superior supercapacitive performance *via* synergizing the interfacial bonding and interlayer spacing. Nano Lett. **23**(12), 5663–5672 (2023). <https://doi.org/10.1021/acs.nanolett.3c01307>
10. M.M. Ovhal, H.B. Lee, V.V. Satale, B. Tyagi, S. Chowdhury et al., One-meter-long, all-3D-printed supercapacitor fibers based on structurally engineered electrode for wearable energy storage. Adv. Energy Mater. **14**(6), 2303053 (2024). <https://doi.org/10.1002/aenm.202303053>
11. Q. Liu, A. Zhao, X. He, Q. Li, J. Sun et al., Full-temperature all-solid-state Ti3C2Tx/aramid fiber supercapacitor with optimal balance of capacitive performance and flexibility. Adv. Funct. Mater. **31**(22), 2010944 (2021). <https://doi.org/10.1002/adfm.202010944>
12. L. Wang, M. Zhang, B. Yang, J. Tan, Lightweight, robust, conductive composite fibers based on MXene@Aramid nanofibers as sensors for smart fabrics. ACS Appl. Mater. Interfaces **13**(35), 41933–41945 (2021). <https://doi.org/10.1021/acsami.1c13645>
13. K.A.S. Usman, J. Zhang, S. Qin, Y. Yao, P.A. Lynch et al., Inducing liquid crystallinity in dilute MXene dispersions for facile processing of multifunctional fibers. J. Mater. Chem. A **10**(9), 4770–4781 (2022). <https://doi.org/10.1039/d1ta09547a>
14. C. Zhu, F. Geng, Macroscopic MXene ribbon with oriented sheet stacking for high-performance flexible supercapacitors. Carbon Energy **3**(1), 142–152 (2021). <https://doi.org/10.1002/cey2.65>
15. S. Li, Z. Fan, G. Wu, Y. Shao, Z. Xia et al., Assembly of nanofluidic MXene fibers with enhanced ionic transport and capacitive charge storage by flake orientation. ACS Nano **15**(4), 7821–7832 (2021). <https://doi.org/10.1021/acsnano.1c02271>
16. X. Cao, G. Wu, K. Li, C. Hou, Y. Li et al., High-performance Zn^2+^-crosslinked MXene fibers for versatile flexible electronics. Adv. Funct. Mater. **34**(46), 2407975 (2024). <https://doi.org/10.1002/adfm.202407975>
17. J. Zhang, S. Uzun, S. Seyedin, P.A. Lynch, B. Akuzum et al., Additive-free MXene liquid crystals and fibers. ACS Cent Sci **6**(2), 254–265 (2020). <https://doi.org/10.1021/acscentsci.9b01217>
18. W. Eom, H. Shin, R.B. Ambade, S.H. Lee, K.H. Lee et al., Large-scale wet-spinning of highly electroconductive MXene fibers. Nat Commun **11**(1), 2825 (2020). <https://doi.org/10.1038/s41467-020-16671-1>
